# Validation and implementation of a method for microarray gene expression profiling of minor B-cell subpopulations in man

**DOI:** 10.1186/1471-2172-15-3

**Published:** 2014-01-31

**Authors:** Kim Steve Bergkvist, Mette Nyegaard, Martin Bøgsted, Alexander Schmitz, Julie Støve Bødker, Simon Mylius Rasmussen, Martin Perez-Andres, Steffen Falgreen, Anders Ellern Bilgrau, Malene Krag Kjeldsen, Michael Gaihede, Martin Agge Nørgaard, John Bæch, Marie-Louise Grønholdt, Frank Svendsen Jensen, Preben Johansen, Karen Dybkær, Hans Erik Johnsen

**Affiliations:** 1 Department of Haematology, Aalborg University Hospital Science and Innovation Center, Sdr Skovvej 15, DK-9000 Aalborg, Denmark; 2 Department of Biomedicine, Aarhus University, Aarhus, Denmark; 3 Department of Mathematical Sciences, Aalborg University, Aalborg, Denmark; 4 Department of Otolaryngology, Head & Neck Surgery, Aalborg University Hospital, Aalborg, Denmark; 5 Cardiothoracic Surgery, Aalborg University Hospital, Aalborg, Denmark; 6 Clinical Immunology, Aalborg University Hospital, Aalborg, Denmark; 7 Vascular Surgery, Aalborg University Hospital, Aalborg, Denmark; 8 Abdominal Surgery, Aalborg University Hospital, Aalborg, Denmark; 9 Pathology, Aalborg University Hospital, Aalborg, Denmark; 10 The Clinical Cancer Research Center, Aalborg University Hospital, Aalborg, Denmark; 11 The Department of Clinical Medicine, Aalborg University, Aalborg, Denmark

**Keywords:** Microarray gene expression profiling, RNA purification, B-cell subpopulations, Fluorescence activated cell sorting

## Abstract

**Background:**

This report describes a method for the generation of global gene expression profiles from low frequent B-cell subsets by using fluorescence-activated cell sorting and RNA amplification. However, some of the differentiating compartments involve a low number of cells and therefore it is important to optimize and validate each step in the procedure.

**Methods:**

Normal lymphoid tissues from blood, tonsils, thymus and bone marrow were immunophenotyped by the 8-colour Euroflow panel using multiparametric flow cytometry. Subsets of B-cells containing cell numbers ranging from 800 to 33,000 and with frequencies varying between 0.1 and 10 percent were sorted, subjected to mRNA purification, amplified by the NuGEN protocol and finally analysed by the Affymetrix platform.

**Results:**

Following a step by step strategy, each step in the workflow was validated and the sorting/storage conditions optimized as described in this report. *First,* an analysis of four cancer cell lines on Affymetrix arrays, using either 100 ng RNA labelled with the Ambion standard protocol or 1 ng RNA amplified and labelled by the NuGEN protocol, revealed a significant correlation of gene expressions (r ≥ 0.9 for all). Comparison of qPCR data in samples with or without amplification for 8 genes showed that a relative difference between six cell lines was preserved (r ≥ 0.9). *Second,* a comparison of cells sorted into PrepProtect, RNAlater or directly into lysis/binding buffer showed a higher yield of purified mRNA following storage in lysis/binding buffer (p < 0.001). *Third,* the identity of the B-cell subsets validated by the cluster of differentiation (CD) membrane profile was highly concordant with the transcriptional gene expression (p-values <0.001). *Finally,* in normal bone marrow and tonsil samples, eight evaluated genes were expressed in accordance with the biology of lymphopoiesis (p-values < 0.001), which enabled the generation of a gene-specific B-cell atlas.

**Conclusion:**

A description of the implementation and validation of commercially available kits in the laboratory has been examined. This included steps for cell sorting, cell lysis/stabilization, RNA isolation, RNA concentration and amplification for microarray analysis. The workflow described in this report will enable the generation of microarray data from minor sorted B-cell subsets.

## Background

Haematological malignancies are characterised by a continuous sub-clonal selection that becomes abnormally and non-homogeneously distributed within individual tumours. In most cases, malignant transformation and metastases are clonal since they are derived from single cells that, at least initially, preserve many features of the hierarchical structure of the normal tissue of origin. However, this has not been analysed systematically for malignancies [1-3]. Global gene expression profiling (GEP) and genetic alterations have already resulted in changes in the classification of malignant B-cell disorders [4-8], including identification of the normal cell of origin. Consequently, highly pure normal subpopulations are essential in order to investigate the complex cellular and molecular mechanisms involved in the stepwise B-cell differentiation in normal and malignant conditions [9-11].

The molecular mechanisms that control B-cell lymphopoiesis are regulated by the coordinated activity of a group of so-called master regulatory transcription factors (TF) [12]. TFs can be divided into those that maintain B-cell lineage commitment (such as PAX5) and the TFs involved in the germinal centre (GC) reaction (such as BACH2 and BCL6), and those that promote and facilitate end-stage differentiation, notably IRF4, PRDM1, and XBP1. This ensures the separation of a range of well-defined subsets including pre-BI cells, pre-BII cells, immature (I), naive (N), centrocytes (CC), centroblasts (CB), memory B-cells (M), plasmablasts (PB) and end-stage antibody producing plasma cells (PC) [13-15].

A limiting factor in studies of B-cell subsets, however, is that some of the differentiating compartments involve a low number of cells and it is therefore important to optimize and validate each step in the procedure for global GEP of minor B-cell subsets following fluorescence-activated cell sorting (FACS). In addition, the conventional labelling kits require an input of 50 – 500 ng of total RNA, which corresponds to around 10^5^ to 10^6^ cells, depending on cell type. However, it is practically impossible to obtain such numbers from some B-cell subpopulations as they only constitute between 0.1 and 10% of the lymphoid tissue [16].

Early human B-cell development has been characterized by GEP, using FACS purified B-cell subsets in bone marrow (BM) [17,18]. However, whereas the early B-cell differentiation has been characterized, the simultaneous sorting of post-germial centre B-cells in lymphoid tissue has been explored in less detail. The *concept* behind the present project is that a detailed workflow for the generation of GEP from minor B-cell subsets will allow us to establish a B-cell specific gene atlas. This will increase our knowledge of the B-cell differentiation and, ultimately, to use these gene lists in post-GC disease classification. The aims of the study were to validate and implement a fast and efficient method for isolation and generation of GEP from B-cell subsets in peripheral blood, tonsils, thymus and BM in order to generate a gene-specific B-cell atlas. The strategy omits an immunomagnetic purification step for B-cell enrichment before FACS, as often performed [17-20], which is problematic when low frequent B-cells are sorted.

## Methods

### Protocol overview

A flow chart of the established protocol and methods for sorting the B-cell subsets in different tissue [see Additional file 1].

### Impact of amplification

#### Gene specific amplification determined by qPCR

Total RNA from one million cells was extracted from six multiple myeloma (MM) cancer cell lines (CCLs) (MOLP-8, KMS-12-BM, RPMI-8226, OPM-2, LP-1, and KMM-1) using RNAeasy Plus Micro equipment (QIAGEN, Hilden, Germany). Genomic DNA was removed using gDNA Eliminator Spin Columns (QIAGEN, Hilden, Germany). RNA quality was evaluated with an Agilent 2100 bioanalyzer (Agilent Technologies, Inc., Palo Alto, CA) (RIN > 9.8). Total RNA from each CCL was processed in parallel by either directly converting 500 ng to cDNA (non-amplified) with SuperScript III First-Strand Synthesis Supermix (Invitrogen, Paisley, UK) or by amplifying 5 ng with an Ovation Pico WTA system (NuGEN Technologies, Inc., San Carlos, CA), as described by the manufacturer. QPCR assays were performed by comparing amplified cDNA at 25 ng/reaction to non-amplified cDNA derived from SuperScript III at 25 ng/reaction (total RNA equivalents). Commercially available Taqman primer probes sets, previously described in the qPCR Section, were used.

#### Comparing NuGEN protocol to standard protocol

Total RNA from one million cells of the same four CCLs KMM-1, OPM-2, U2932_M, and SU-DHL-5 was subjected to the NuGEN protocol or to the standard protocol from Ambion (Ambion WT Expression kit, Ambion, Inc., Austin, TX) following the manufactures recommendations. The input of total RNA was 1 ng for the NuGEN protocol whereas the input for the Ambion protocol was 100 ng.

### Optimisation of storage buffer

#### Selection of storage buffer for sorted cells

The storage buffer was examined by sorting 15,000 fresh naive tonsil cells from a single donor directly into 12 separate tubes containing 450 μl of either lysis/binding buffer, RNAlater (Ambion, Austin, TX) or PrepProtect. mRNA was isolated using the μMACS™ technology (Miltenyi Biotech, Bergisch-Gladbach, Germany), allowing isolation on μ Columns and elution with pure water. This technology is referred to as magnetic bead isolation (MBI). MBI purification from sorted cells was performed in triplicates, either directly after cell sorting or after 14 days of storage of cells in the various RNA extraction buffers at 4°C, -20°C or -80°C. Before purification, all samples were equilibrated to RT and cells in RNAlater and PrepProtect was recovered from the storage solution by a 5 minute centrifugation (RT) at 5000 g and re-suspended in 450 μl of lysis/binding buffer, following MBI purification. Elutes were stored at -80°C before examining the yields with the TaqMan pre-developed endogenous control assay *PPIA* (333763 F) by RT-qPCR.

### Ethical statement and tissue preparation

All samples were collected following informed consent in accordance with the research protocol accepted by the Ethics Committee for the North Denmark Region (N-20080062MCH). The cells were either FACS sorted fresh (i.e. samples collected, processed and sorted within the same day), or vital cryopreserved for storage and thawed before sorting.

### Isolation of cells from tissues

The isolation strategy of all tissues is described in the following section, including the number of donors (n) for each tissue and the frequency of sorted cells used for generating GEP and establishing a B-cell atlas from different B-cell subsets in tonsils and BM.

#### Peripheral blood mononuclear cells (PBMNC) (frequency of sorted cells: 4,500 – 130,000)

PBMNC (n = 6 ) were isolated by diluting the peripheral blood sample 1:1 in phosphate-buffered saline (PBS) and placing the sample in a LeucoSep tube (Greiner Bio-One, Frickenhausen, Germany), according to the manufacturer’s instructions. The mononuclear cells (MNCs) were washed once and the red blood cells were lysed by adding 9 ml of Easylyse (DAKO, Glostrup, Denmark) to the pellet and incubating the sample for 15 minutes at room temperature (RT). The MNCs were washed twice in PBS and sorted fresh.

#### Tonsils (frequency of sorted cells: 5,600 – 20.000)

Tonsils (n = 8) were obtained by routine tonsillectomy as previously described [9]. In brief, tonsils were placed on ice until homogenization in cold RPMI medium 1640 (Gibco, Invitrogen, UK) using a Medimachine (cod. 79200, Dako, Glostrup, Denmark) with a 35 μM sterile medicon. The cell suspension was passed through a 40 μM filter to remove debris and aggregates. The cell suspension was diluted in PBS before MNCs were isolated using Ficoll-Paque Plus (GE Health Care, Uppsala, Sweden) according to the manufacturer’s instructions. The cells were vital cryopreserved.

#### Thymus (frequency of sorted cells: 1,000 – 33,000)

Thymus (n = 7) was obtained from patients undergoing cardiac surgery and placed on ice immediately after removal and cut into smaller pieces. BM was simultaneously obtained from the same patient and processed as described in the following section. The Thymus tissue was squeezed and chopped with a tweezer in PBS. The cells were washed once in PBS and MNCs were purified using Ficoll-Paque Plus and sorted fresh.

#### BM (frequency of sorted cells: 800 – 25,000)

BM (n = 7) from sternum was obtained from patients undergoing cardiac surgery by physical scraping and scooping of the sternum and placed on ice immediately after removal. The BM was homogenized in 2 ml PBS using a syringe. The red blood cells were lysed by adding 20 ml of Easylyse (DAKO, Glostrup, Denmark) and incubating the sample for 45 min at RT. The samples were washed once in PBS before passing through a 40 μM filter to remove debris and aggregates and sorted fresh.

#### Human malignant B-cell lines

CCLs originating from MM MOLP-8, KMS-12-BM, RPMI-8226, LP-1, OPM-2 and diffuse large B-cell lymphoma (DLBCL) SU-DHL-5 were purchased from DSMZ [German Collection of Microorganisms and Cell Cultures, Braunschweig, Germany], whereas the MM CCL KMM-1 was purchased from JCRB [Japanese Collection of Research Bioresources Cell Bank, Japan]. DLBCL CCL U2932_M was generously provided by Jose A Martinez-Climent [Molecular Oncology Laboratory, University of Navarra Pamplona, Spain].

### Multiparametric flow cytometry

MNCs were washed twice in PBS and in the final wash 2% BSA was added. The cells were stained with a 6–8 colour panel of monoclonal antibodies (mAbs). Isolating the B-cell subsets from PBMMC, tonsils, thymus and BM had the following five mAbs in common: CD20 clone 2H7 conjugated with pacific blue (eBioscience, San Diego, CA), CD45 clone 2D1 conjugated with anemonia majano cyan (BD Biosciences, San Jose, CA), CD10 clone HI10a conjugated with phycoerythrin/cyanin7 (BD Biosciences, San Jose, CA), CD27 clone L128 conjugated with Allophycocyanin (BD Biosciences, San Diego, CA), CD38 clone HIT2 conjugated with Alexa Flour 700 (ExBio Vestec, Czech Republic). CD19 conjugated with PERCPCy5.5 (BD Biosciences, San Diego, CA) were included in all samples except in tonsils. In BM, CD34 conjugated with phycoerythrin (BD Biosciences, San Diego, CA) were included. In tonsils, CD3 clone SK7 conjugated with fluorescein isothiocyanate (BD Biosciences, San Jose, CA), CD44 clone IM7 conjugated with peridinin chlorophyll protein/cyanin 5.5 (eBiosciences, San Diego, CA), and CXCR4 clone 12G5 conjugated with phycoerythrin (Beckman coulter, Brea, CA) were included. In thymus, sIgM conjugated with FITC (DAKO, Carpinteria, US) and sIgG conjugated with phycoerythrin (SouthernBiotech, Alabama, US) were included.

#### CD marker combinations used to distinguish between the B-cell subsets [see Additional file 1]

Briefly, all cells but tonsils were incubated for 30 minutes at room temperature (RT) (tonsils on ice) in darkness. Sorting of B-cell subsets was performed using a FACSAria2 cell sorter (BD Biosciences, San Jose, CA) at RT. Compensation was automatically calculated by FACSDiva software using single-stained control samples with the mAbs previously listed. Immediately before acquisition, the cells were filtered through a 35-μm filter (Cell Stainer, BD Biosciences). The purity of the isolated B-cell subsets (>90%) was confirmed by sorting approximately 1,000 cells into PBS and reacquisition of the sorted B-cell subsets. The cells were sorted into 450 μl of lysis/binding buffer (Miltenyi Biotech, Bergisch-Gladbach, Germany), except for the first PBMNC processed, which were sorted into 1 ml of PrepProtect (Miltenyi Biotech, Bergisch-Gladbach, Germany). The sorted B-cell subsets were stored at -20°C.

### Global GEP of sorted B-cell subsets

Gene expression analysis of FACS sorted cells was performed using the Gene Chip Human Exon 1.0 ST (Exon) or the Gene chip HG U133 Plus 2.0 (U133) arrays (Affymetrix, Santa Clara, CA). In brief, mRNA was isolated by MBI and the eluted mRNA was concentrated to 5–10 μl by a volume reduction step using a speedVac Concentrator 5310 (Eppendorf, Hamburg, Germany). Five μl was used as input for amplification with the Ovation Pico WTA system (NuGEN Technologies, Inc., San Carlos, CA) following the manufacturer’s protocol. Due to technical challenges, it was not possible to determine the concentration of mRNA derived from five μl of elute. Finally, the samples were hybridised to the Exon or U133 array. This is referred to as the NuGEN protocol.

### Reverse transcription and qPCR

#### Complementary DNA synthesis

Five μl of mRNA from sorted cells or 500 ng of total RNA from CCLs were used in cDNA synthesis using SuperScript III First-Strand Synthesis Supermix (Invitrogen, Paisley, UK). Random hexamers (50 μM) and oligo(dT) (50 μM) primers were used in a final volume of 20 μl.

#### qPCR

qPCR was performed on a LightCycler 480 II using the LightCycler 480 Probes Masters PCR mix (Roche Diagnostics, Hvidovre, Denmark) and Taqman gene expression assays (Applied Biosystems, Foster City, CA) in a final reaction volume of 20 μl. Water controls, no-RT samples, and inter-run calibrator samples were included on all plates. Commercially available Taqman primer/probe sets were used (Applied Biosystems, Foster City, CA) for three endogenous control genes: *PPIA* [4333763 F], *GAPDH* [4333764 F] and *TBP* [4333769 F], for three transcription factors involved in the late B-cell lymphopoiesis: *IRF4* [Hs01056534_m1], *PRDM1* [Hs00153357_m1] and *XBP1* [Hs00231936_m1] and two genes related to the oncogenesis of MM: *MGST1* [Hs00220393_m1] and *WHSC1* alias *MMSET* [Hs00983716_m1].

### Statistical analysis

All data processing and statistical analysis was performed using Affymetrix GeneChip Command Console Software (AGCC), Partek Genomics Suite version 6.5 (Partek Inc., St. Louis, MO, USA), built-in Excel macros, and the statistical software system R, version 2.15.1. For all tests, p-values below 5% (p < 0.05) were considered statistically significant.

#### Gene specific amplification determined by qPCR

For a specific gene, x, the difference in Cq values (dCq) between two amplified CCLs, 1 and 2, (Cq_x,1_ – Cq_x,2_)_amplified,_ was plotted against the dCq-value for the same two non-amplified cell lines (Cq_x,1_ – Cq_x,2_)_non-amplified_. Pearson’s correlation coefficient between the two dCq values was calculated. A significant positive Pearson’s correlation coefficient indicates preservation of differential gene expression after amplification [21]. In addition, for each gene, the six cell lines were ranked from high to low expression. This ranking was performed for both the non-amplified and the amplified cell lines, and compared using Spearman’s rank correlation. A test for inconsistent ranking was carried out by an exact permutation test.

#### Selection of storage buffer for sorted cells

The Cq values were analysed by a 2-way analysis of variance (ANOVA) with the two factors storage buffer at three levels (Lysis buffer; PrepProtect; RNAlater) and condition at four levels (Directly; 4°C; -20°C; -80°C).

#### Data processing and analysis

CEL-files from the two array types U133 and Exon were generated by AGCC [for results of the initial quality control analyses, see Additional file 2]. CEL-files were imported into Partek and RMA normalised. Due to in-homogenous variance, the difference in expression values between the B-cell subsets for each of the endogenous control genes was tested by a Kruskal-Wallis one-way analysis of variance. Furthermore, for biological validation, the expression values of selected genes with well-known function in each B-cell subset of the BM were analysed. Significant expressions between functional classes of B-cell subsets were tested by a two-tailed *t*-test with unequal variance. Correlations between two measurements were evaluated throughout by the Pearson’s correlation coefficient and denoted by an r. Finally, plots of gene differences versus gene averages, MA plots, were used to examine the concordance between methods for generating global GEP.

#### Concordance between the pre-defined CD marker and array based transcript expressions

The B-cell subsets were divided into two groups based on the pre-defined positive or negative CD marker. The mean and standard deviations were calculated for the positive and negative groups based on the gene expression value for the CD marker. Discordance was registered if a gene expression value for a B-cell subset was observed in the opposite group as defined by the CD marker. Fisher’s exact test for 2x2 tables was used to test for independence between the groupings based on CD marker and GEP [see Additional file 3].

#### Freshly sorted samples or cryopreservation before sorting

The ratios of excluded samples were compared for freshly sorted samples as well as for those cryopreserved before sorting. Samples were either excluded due to low amplification yield or unsatisfactory quality control parameters on array. The significance between the ratios was tested by a chi-square test for equality of proportions.

## Results

### Impact of amplification

#### Gene specific amplification determined by qPCR

**Figure 1:**
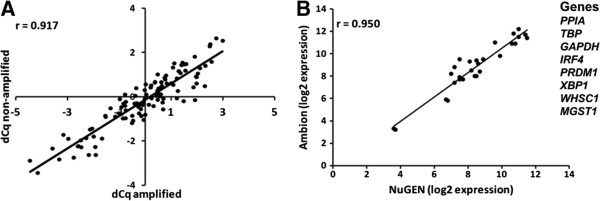
**Evaluating the impact of amplification. A**: Gene-specific amplification determined by qPCR. Scatter plot comparing dCq for a gene between two cell lines for non-amplified samples with dCq for the same gene and cell lines after amplification. Pearson’s correlation coefficient is r = 0.917. Genes included *PPIA*, *TBP*, *GAPDH*, *IRF4*, *PRDM1*, *XBP1*, *WHSC1* and *MGST1*. **B**: Comparing NuGEN protocol to standard protocol. Scatter plot comparing the Exon expression values generated by the NuGEN protocol to expression values generated by the Ambion protocol. Genes included are *PPIA*, *TBP*, *GAPDH, IRF4*, *PRDM1*, *XBP1*, *WHSC1,* and *MGST1*. The Pearson’s correlation coefficient is r = 0.950.

A major concern is the amplification method and how it affects the ability to maintain the relative amount of the starting RNA, the so called fidelity of amplification [22]. This was evaluated by comparing the expression of eight genes, which included the three endogenous control genes (*PPIA, TBP* and *GAPDH*), three transcription factors involved in the late B-cell lymphopoiesis (*XBP1, IRF4* and *PRDM1*)*,* and two genes related to oncogenesis of MM (*MGST1 and WHSC1*) in six amplified and non-amplified CCLs [see Additional file 4]. Total RNA from one million cells was extracted from each of the six CCLs. The ranks of the selected genes in non-amplified and amplified CCLs were fairly preserved (p-values = 0.02, 0.10, 0.05, 0.01, 0.07 and 0.03 for CCLs KMM-1, KMS-12-BM, LP-1, MOLP-8, OPM-2 and RPIM-8226, respectively). However, a transcript-specific amplification effect was noticed, meaning that some transcripts were amplified with a higher efficiency than others, exemplified in *PPIA* compared to *WHSC1* [see Additional file 5]. We therefore examined whether the relative difference in gene expression was preserved in the six CCLs after amplification. For each gene, the difference in delta Cq between two non-amplified CCLs was plotted against the delta Cq in the same amplified CCLs with results as presented in Figure 1A. Pearson’s correlation coefficient (r = 0.92) indicated a good preservation of the gene-specific amplification across samples. These results emphasise that, when this protocol is to be used to generate global gene expression data, the expression level of a specific gene should only be compared across samples that are all non-amplified or all amplified.

#### Comparing NuGEN protocol to standard protocol

The NuGEN’s protocol was compared to Ambion’s protocol, which utilises the most routinely used labelling method. The Ambion protocol is based on *in vitro* transcription (IVT) of a cDNA template into complementary RNA (cRNA), using T7 RNA polymerase [23,24]. The same total RNA, extracted from one million cells, from the four CCLs was processed with either Ambion’s or NuGEN’s protocol, reducing the input amount by a 100 times. Both protocols generated acceptable yields to target preparation and hybridisation to Exon array, according to the manufacturer’s recommendations, as presented in Table 1. The non-specific amplification products, when amplification was performed without RNA (negative control), were nearly the same with 14% (5.2 μg) for Ambion and 11% (0.8 μg) for NuGEN. In addition, we noticed that the present calls were a bit higher for the Ambion protocol.

**Table 1:** Amount of starting material and percent present calls on Exon arrays

**Protocol**	**Sample**	**Starting amount**	**Amount of aRNA/cDNA after processing**	**Present call**
Ambion	KMM-1	100 ng	44.9 μg	61%
	SU-DHL-5	100 ng	29.1 μg	57%
	U2932_M	100 ng	40.8 μg	64%
	OPM-2	100 ng	35.8 μg	61%
	Negative control	0 ng	5.2 μg	
NuGEN	KMM-1	1 ng	7.2 μg	51%
	SU-DHL-5	1 ng	7.1 μg	46%
	U2932_M	1 ng	6.9 μg	51%
	OPM-2	1 ng	7.6 μg	50%
	Negative control	0 ng	0.8 μg	

Total RNA from the same four CCLs was either processed with the Ambion or NuGEN protocol. The input amount for the NuGEN protocol was reduced by 100 times and the amount present calls were calculated with the PLIER algorithm in Expression Console (Affymetrix). Negative control is amplification without total RNA.

The reproducibility between the two protocols was first examined by the narrow set of previously used genes [see Additional file 6]. The expression of the selected genes generated by the NuGEN protocol was plotted against the expression of the genes generated by the Ambion protocol as presented in Figure 1B. We observed a high degree of correlation between the two protocols regarding the expression from the eight genes (r = 0.950). Next, we measured the reproducibility of the gene expression on a global scale by generating MA plots [see Additional file 7]. We observed a Pearson’s correlation coefficient ranging from r = 0.892 to r = 0.904 for all pair-wise comparisons of protocols, demonstrating an acceptable reproducibility and robustness of the NuGEN protocol.

### Optimisation of storage buffer

#### Selection of storage buffer for sorted cells

*A priori*, the handling procedures were considered important, including methods for RNA extractions [25-29]. In one previous study, the best method for RNA extraction from flow-sorted cells was MBI [30], which was selected for the present study. A pilot study revealed that the recovery of relative few cells (50,000-200,000) after centrifugation varied by up to 50% (Unpublished observations). Cells sorted in conventional storage buffers such as PrepProtect or RNAlater include a centrifugation step in order to re-suspend the cells in lysis buffer. Therefore, we wanted to test if the lysis/binding buffer could be used as a storage buffer, thereby omitting the centrifugation step in the procedure.

**Figure 2:**
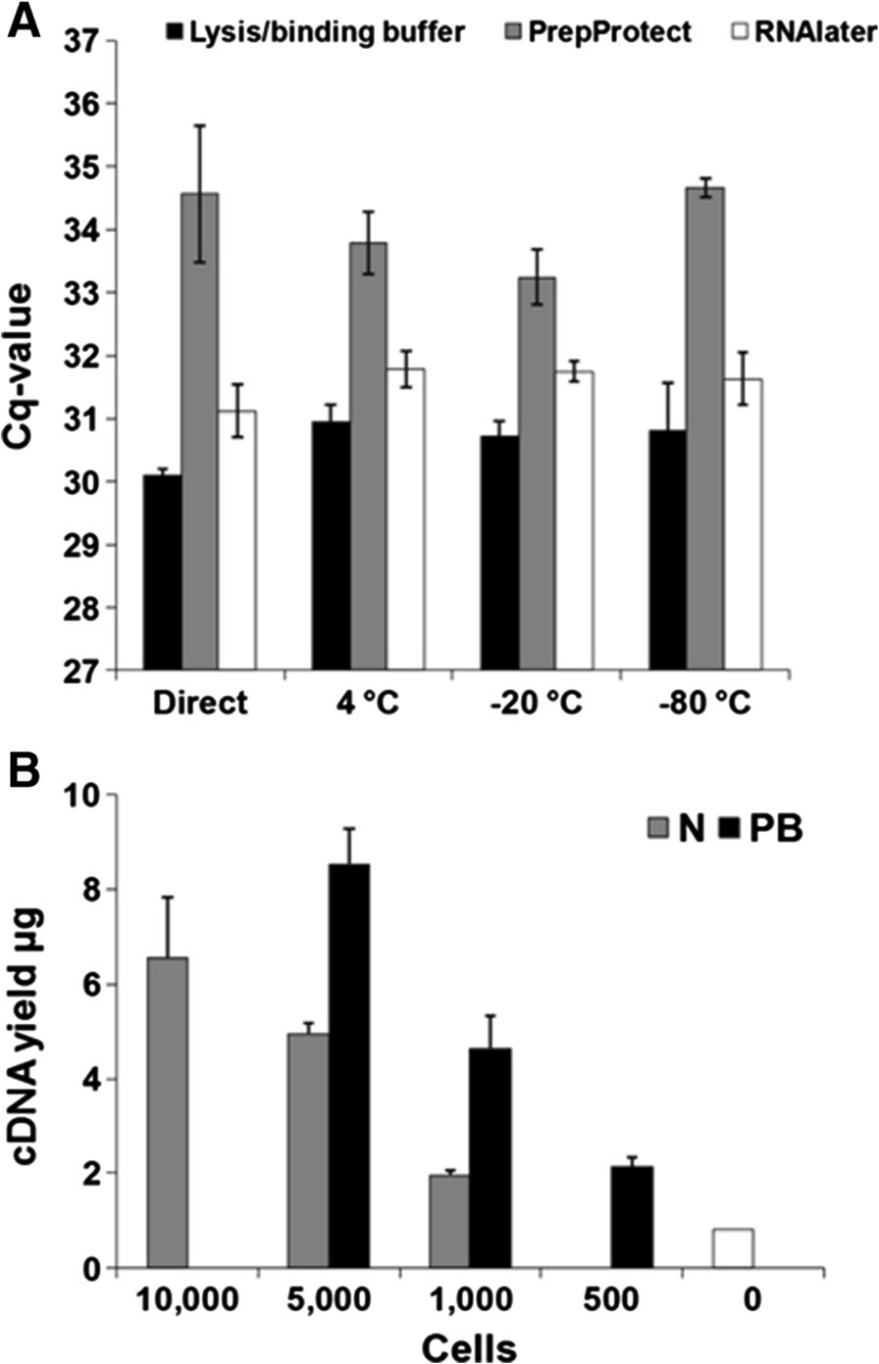
**Optimization and performance of the protocol. A**: Selection of storage buffer for sorted cells. RNA yield from 15,000 N tonsil cells. The cells were sorted into different storage reagents and mRNA was purified with MBI either immediately after cell sorting or after 14 days of storage at 4°C, -20°C and -80°C. RNA yields were determined by RT-qPCR targeting *PPIA*. Mean Cq values were calculated from triplicate RNA extractions from each storage reagent. Error bars represent SD (n = 3). **B**: Amplified cDNA yield from a fixed number of flow sorted N and PB cells derived from a tonsil. The cells were sorted in lysis/binding buffer and mRNA was purified with MBI. Prior to amplification, the mRNA was concentrated by speedVac centrifugation. The amplified cDNA yield was measured on the nanodrop. Error bars represent SD of 3 experiments.

The yield of mRNA from 15,000 sorted cells was tested in three different storage buffers after immediate or postponed time to mRNA purification. The mRNA yields, indicated by Cq values, were significantly higher for cells sorted into lysis/binding buffer compared to PrepProtect (p < 0.001) or RNAlater (p < 0.001), as shown in Figure 2A. In addition, for cells sorted into lysis/binding buffer, mRNA purification directly after cell sorting resulted in a significantly higher yield (p < 0.001) compared to mRNA extraction from cells performed after 14 days of storage at 4°C, -20°C and -80°C.

### Performance of the protocol

#### Amplified yield from FACS tonsil B-cell subsets

The technical variation in the protocol, including flow sorting, freezing, purifying mRNA, up-concentrating RNA and amplifying the samples, was addressed by sorting two B-cell subsets, namely the N and the PB from tonsils in triplicates from a single donor. By sorting different numbers of cells (500, 1,000 and 5,000 PBs and 1,000, 5,000 and 10,000 N cells), we found that the amplified cDNA yield per cell varied depending on cell type, with PBs providing a higher yield compared to N cells (Figure 2B). Based on the amplified cDNA yield, 1,000 PB and 5,000 N was sufficient for generating global GEP, according to the input requirement in the protocol. These samples were only intended to be used to investigate the technical variation in the protocol and not used to generate global GEP.

#### Tissue-independent yield and QC on microarray data

Using the protocol, GEP was successfully established on a low number of FACS sorted cells in the frequency of 800–33,000 cells in lysis/binding buffer from different tissues including PBMNC, tonsils, thymus and BM (Table 2). For PBMNCs, GEP was performed on both fresh (F) and cryopreserved (C) sorted cells with no significant difference in the number of excluded samples (p = 0.991), demonstrating the applicability of the protocol to cryopreserved material irrespective of B-cell subset (manuscript in preparation).

**Table 2:** Tissue independent yield and QC on microarray

	**Protocol**				**Demographic data**		**Array type**
**Tissue/B-cell subsets**	**Sorted cells (x1000)**	**Total no of Amp. samples**	**Excluded**	**Amp. yield cDNA μg**	**Gender**	**Age**	
**PBMNC (F) n = 6**	PreP				5 F; 1 M	49 ± 13	Exon
I	9-43	6	0	5.3 ± 0.9			
N	100-130	6	2	6.5 ± 0.9			
M	100-130	6	0	7.1 ± 0.6			
PB	4.5-29	6	1	5.3 ± 1.3			
**PBMNC (F) n = 3**	Lys				1 F; 2 M	43 ± 17	Exon
I	7.5	3	0	6.6 ± 1.0			
N	7.5	3	0	7.1 ± 1.1			
M	7.5	3	0	8.6 ± 1.5			
PB	4.5-7.5	3	0	9.3 ± 0.3			
**PBMNC (C) n = 3**	Lys				Identical		
I	7.5	3	0	6.0 ± 1.5			
N	7.5	3	0	7.3 ± 1.1			
M	7.5	3	0	6.4 ± 1.2			
PB	4.5-7.5	3	1	9.8 ± 0.5			
**Tonsils (C) n = 8**	Lys				6 F; 2 M	17 ± 10	U133
N	10-25	8	2	6.8 ± 0.8			
CB	10-20	8	1	8.8 ± 0.9			
CC	10-12.5	8	1	8.8 ± 0.9			
M	10-20	8	2	6.7 ± 0.9			
PB	5.6-20	8	1	9.3 ± 0.6			
**Thymus (F) n = 7**	Lys				2 F; 5 M	63 ± 12	Exon
N	5-25	6	2	7.6 ± 2.2			
M IgM	3.5-25	5	1	6.4 ± 3.1			
M IgG	10-33	7	2	8.4 ± 3.1			
PB	1-4.5	6	1	7.2 ± 4.1			
**BM (F) n = 7**	Lys				Identical		
PreBI	3-25	6	0	7.8 ± 2.2			
PreBII	3-25	7	1	7.8 ± 1.2			
I	0.8-25	7	2	6.0 ± 1.7			
N	8-25	7	0	6.3 ± 2.2			
M	2-25	7	1	7.1 ± 2.5			
PC	7.5-25	7	1	8.0 ± 2.2			

The protocol was applied to PBMNC, tonsils, thymus and BM. The samples were sorted from either fresh (F) or cryopreserved (C) cells into PreProtect (PreP) or lysis/binding buffer (Lys). Excluded samples were either due to failed amplification or QC on array.

#### Concordance between the predefined CD marker and array-based transcript expressions

A quality control step was included to validate the identity of the sorted B-cell subsets by correlating the pre-defined surface expressed CD markers used for FACS of the B-cell subsets to the CD markers transcript expression levels on microarray [see Additional file 3]. In summary, there was a highly significant (p < 0.001) concordant expression of CD protein markers and transcript expressions. For example, in a total of 44 PBMNC samples concordance was observed for CD20, CD10, CD27, and CD38 gene expression in 43, 43, 42, and 41 samples respectively [see Additional file 3: Table S2].

### Biological validation

Global GEP data were generated from sorted B-cell subsets in PBMNC and lymphoid tissues. The initial data analysis was carried out to explore similarities and differences between samples and to determine whether the samples could be grouped into distinct B-cell subsets. The data matrix was subjected to principal component analysis (PCA) and the results are shown in the Additional file 8. It is noted that the preBI and II; immature, naive and memory B-cells; plasmablasts and plasma cells tended to cluster in separate biological relevant groups, as expected. In addition, naive and memory B-cells tended to cluster together, regardless of tissue, as did the centroblasts and centrocyte populations in tonsils, regardless of array type. Of note, the tonsil donors on the Exon and U133 arrays are not identical.

**Figure 3:**
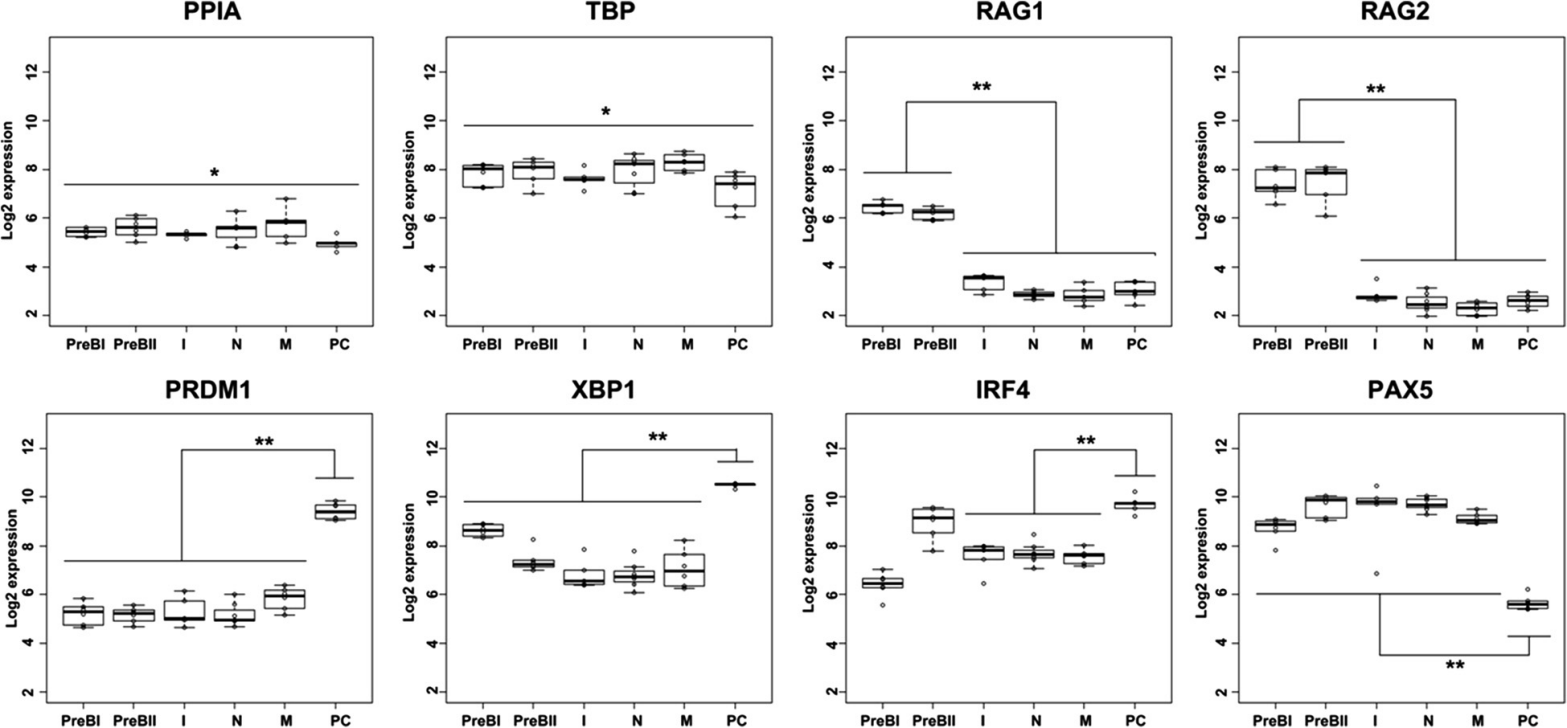
**Biological validation.** The expression values of selected genes in the B-cell subsets derived from the BM on the Exon array. Pre-B: Pre B cells, I: immature B cells, N: naïve B cells, M: memory B cells, PC: plasma cells. *p ≥ 0.05, **p < <0.001.

The usefulness of the protocol was examined in the global GEP data generated from normal BM samples on the Exon array. This was evaluated by comparing the expression of eight genes, which included the former evaluated endogenous control genes (*PPIA* and *TBP*). In addition, the transcription factors involved in the B-cell differentiation (*PAX5*, *XBP1*, *IRF4* and *PRDM1*) and the genes *RAG1*, *RAG1*, involved in the early B-cell differentiation, were included. Dys-regulation of these genes is among the most central mediators of malignant transformation. The box plots of eight genes are presented in Figure 3. As expected, the endogenous control genes *PPIA* and *TBP* were not differentially expressed in the six B-cell subsets (p ≥ 0.05) [31]. *RAG1* and *RAG2*, that initiate V(D)J recombination in the BM, and are essential for the maturation of pre-B-cells [32,33], were significantly up-regulated in PreBI cells and PreBII cells compared to the other B-cell subsets. The TF PAX5, which is essential for early commitment to the B-cell lineage and maintained in all B-cell subsets, except in PC [34], was significantly up-regulated in the five B-cell subsets compared to the PC. The TFs PRDM1, XBP1 and IRF4, which promote and facilitate PC differentiation [12], were significantly up-regulated in PC. In addition, IRF4 were significantly down-regulated in PreBI cells (p < 0.001) and significantly up-regulated in PreBII cells (p = 0.003) compared to I, N, and M B-cell subsets. In line with these findings, it has been shown that IRF4 promotes PreB-cell differentiation by inducing conventional light-chain gene transcription and rearrangement [35].

Likewise, in the tonsil data set, eight evaluated genes were expressed in accordance with the biology of lymphopoiesis (p-values < 0.001) [see Additional file 9]. The genes included the formerly evaluated transcription factors *PAX5*, *PRDM1* and *IRF4*. In addition, four genes (BCL6, AICDA, BACH2 and CXCR4) involved in the GC reaction and the proliferation marker MKI67 were included. Finally, with the implemented and validated protocol, a gene specific B-cell atlas was generated from the BM and tonsil tissue [see Additional files 10 and 11].

## Discussion

The aim of this project was to implement and validate the steps in a protocol for generating global GEP of FACS sorted low frequent B-cell subsets in lymphoid tissue and blood. In the present study, we have validated a protocol for microarray studies of minor subsets and sorted cells from different tissues in the range 800 to 33,000 cells into lysis/binding buffer.

In order to use GEP for the analysis of populations below 100,000 cells, RNA has to be reverse-transcribed and amplified. The amplification technologies have been recently evaluated with respect to reproducibility and sensitivity and it was observed that the NuGEN protocol was the most suitable for amplification of pico amounts of RNA [36]. In addition, a study comparing different amplification protocols, including NuGEN protocol, Message Amp (Ambion), Small Sample Target Labelling Assay Version II (Affymetrix) and BioArray Small Sample Amplification Protocol (Enzo Life Sciences) found that all technologies performed acceptably. The NuGEN protocol, however, resulted in the most sensitive and specific data when 10 ng of RNA was amplified [37].These studies were the basis for choosing the NuGEN protocol in this study. The amplification by the NuGEN protocol was first evaluated by comparing the expression of eight genes in six non-amplified and amplified CCLs. We noticed a gene-specific amplification, meaning that some sequences or parts of transcripts were amplified better than others. However, differential gene expression was preserved across the CCLs (r = 0.917), which is in line with previous findings [21]. Next we compared the NuGEN protocol to the standard protocol from Ambion by examining the generated global gene expression data on the Exon array. The reproducibility was good, both when the expression was compared globally (r = 0.892 to 0.904) and compared for a narrow set of genes (r = 0.950). Correlation coefficients between methods are usually lower than 0.90 when compared [22].

By sorting cells directly into lysis/binding buffer, a significantly higher yield was obtained both for direct and postponed time for preparation of mRNA purification. The reduced yields obtained from cell storage in RNAlater are very likely due to the centrifugation step introduced to recover the cells prior to lysis, but do not explain the higher Cq values of approximately 3–4 obtained in PrepProtect. Of notice, reducing the centrifugation force to 3000 g the same Cq values was obtained for cells stored in PrepPretect; however, any inhibition in the enzymatic assay has not been tested, which also may explain the poor yield.

By using the NuGEN amplification method and MBI technology, we obtained sufficient amplified cDNA yields from 1,000 PB and 5,000 N B-cells to ensure successful microarray analysis.

As the present work represents one of the first global transcriptome studies with sorted low frequent B-cell subsets, the reliability of the results obtained has been considered a critical issue. Certainly, contamination of the sorted B-cell subsets with “rosetting” non B-cells cannot be excluded. However, it is evident from the results obtained in the present analysis that such contaminations do not compromise gene expression analysis. First, the CD markers used for identification and sorting are concordantly expressed at the transcript level [see Additional file 5]. Secondly, biological validation of the generated GEP was conducted in the BM and tonsil samples for eight genes and we found them to be expressed in accordance with our knowledge of the B-cell lymphopoiesis.

## Conclusion

A protocol for microarray analysis of FACS-sorted low frequent B-cells was implemented and validated to achieve new insights into B-cell differentiation. The protocol consisted of CD panels for sorting early/mature and post-GC B-cells in BM, tonsils, thymus and blood; sorting cells directly into lysis/binding buffer; purifying mRNA by MBI; and concentrating RNA by speedVac concentrator following amplification with NuGEN’s technology. This protocol represents a significant advance over established protocols by allowing the possibility to sort the cells directly into lysis/binding buffer, which also can be used for long-term storage. In addition, complete CD panels for the simultaneous sorting of pre-GC, GC and post-GC B-cells in lymphoid tissues and blood is provided. A gene specific B-cell atlas was generated in BM and tonsils and a future goal is to assign these profiles in post-GC disease classification by “cell of origin”.

## Abbreviations

GEP: Global gene expression profiling; GC: Germinal centre; TF: Transcription factors; I: Immature; N: Naive; CC: Centrocytes; CB: Centroblasts; M: Memory B-cells; PB: Plasmablasts; PC: Plasma cells; FACS: Following fluorescence-activated cell sorting; PBMNC: Peripheral blood mononuclear cells; BM: Bone marrow; MNCs: Mononuclear cells; CCLs: Cancer cell lines; MM: Multiple myeloma; DLBCL: Diffuse large B-cell lymphoma; mAbs: Monoclonal antibodies; RT: Room temperature; Exon: Gene Chip Human Exon 1.0 ST; U133: Gene chip HG U133 Plus 2.0; MBI: Magnetic bead isolation; AGCC: Affymetrix GeneChip Command Console Software; r: Pearson’s correlation coefficient; ANOVA: Analysis of variance; Cq: Quantification cycles; CD: Cluster of differentiation; IVT: *in vitro* transcription; cRNA: Complementary RNA; F: Fresh; C: Cryopreserved; Lys: Lysis/binding buffer; PCA: Principal component analysis.

## Competing interests

The authors declare that they have no competing interests.

## Authors’ contribution

KSB participated in the design of the study, carried out the flow sorting of cells, purifying mRNA, establish GEP from B-cells, data interpretation and drafted the manuscript. MN participated in the design of the study, data interpretation and drafted the manuscript. MB participated in the design of the study, performed statistical analysis, data interpretation and drafted the manuscript. AS carried out the flow sorting of cells, data interpretation and drafted the manuscript. JSB participated in the design of the study, data interpretation and drafted the manuscript. SMR participated in the design of the study, data interpretation and drafted the manuscript. MPA participated in the design of the study, data interpretation and drafted the manuscript. SF participated in the design of the study, performed statistical analysis, data interpretation and drafted the manuscript. AEB participated in the design of the study, performed statistical analysis, data interpretation and drafted the manuscript. MKK participated in the design of the study, data interpretation and drafted the manuscript. MG participated in data interpretation and drafted the manuscript. MAN participated in data interpretation and drafted the manuscript. JB participated in data interpretation and drafted the manuscript. MLG participated in data interpretation and drafted the manuscript. FSJ participated in data interpretation and drafted the manuscript. PJ participated in data interpretation and drafted the manuscript. KD participated in the design of the study, data interpretation and drafted the manuscript. HEJ participated in the design of the study, data interpretation and drafted the manuscript. All authors read and approved the final manuscript.

## Supplementary Material

Additional file 1 **Flow chart. Procedure and methods used for establishing GEP from FACS-sorted B-cells on the Exon or U133 array.** This included steps for cell sorting, cell lysis/stabilization and storage, RNA isolation, RNA concentration and amplification for microarray analysis. In addition, the CD marker combinations used to distinguish between the B-cell subsets are included. Click here for file

Additional file 2 **Initial data analysis of quality. Table S1.** Initial data analysis of the quality and technical performance of the microarrays was performed using the AGCC. For the tonsils on the U133 arrays, .CEL-files were normalized with MAS5.0. Scaling factor (SF), percentage of present calls (%P), and signal ratios of probe sets interrogating different segments (3′/5′) were comparable across samples, with similar variation among samples of the various B-cell subsets. For the Exon arrays, .CEL-files were normalized with PLIER and %P was evaluated for each tissue ( PBMNC (fresh) mean 53 ± 7; %P thymus mean 51 ± 5; %P BM mean 50 ± 6). Further analysis was performed by evaluating the histogram and signal box plots of the signals. Click here for file

Additional file 3 **Concordance between the pre-defined CD marker and array based transcript expressions.** The B-cell subsets were divided into two groups based on the pre-defined positive or negative CD marker. The mean and standard deviations were calculated for the positive and negative groups based on the gene expression value for the CD marker. Discordance was noticed if the gene expression values for a B-cell subset in the opposite group as a pre-defined CD marker. Fisher’s exact test for 2x2 tables was used to test for independence between the groupings based on CD marker and GEP. **Table S1.** Concordance between the pre-defined CD markers and transcript expression on array in BM. **Table S2.** Concordance between the pre-defined CD markers and transcript expression on array in PBMNC. **Table S3.** Concordance between the pre-defined CD markers and transcript expression on array in thymus. Click here for file

Additional file 4 **Fidelity of amplification. Table S1.** Six CCLs were ranked from high to low expression and normalised to GAPDH. This ranking was performed for both the non-amplified and the amplified CCLs, and compared using Spearman’s rank correlation. A test for inconsistent ranking was carried out by an exact permutation test. Click here for file

Additional file 5 ***PPIA* and *WHSC1* were normalised to *GAPDH* and the gene expression is shown in six non-amplified and amplified CCLs.** The amplification method induces a sequence specific bias, resulting in some sequences or parts of transcripts amplify better than others like *PPIA* compared to *WHSC1.* However, this amplification bias for a gene is preserved across the CCLs. Click here for file

Additional file 6 **Expression of selected genes between two protocols on the Exon array. Table S1.** Expression value of seven selected genes between NuGen and the Ambion protocol on the Exon array. RNA was extracted from the same four CCL (KMM-1, OPM-2, SU-DHL-5 and U2932) and a 100 times less RNA was used as input in the NuGEN protocol compared to Ambion protocol. Click here for file

Additional file 7 **Reproducibility between NuGEN and Ambion protocol. Figure S1.** MA plots were generated for the pair-wise comparisons between CCLs subjected to the NuGEN and Ambion protocol. Exon array signal values were normalized using RMA and Pearson’s correlation coefficient was calculated using the Affymetrix Expression Console analysis package. The log2 fold change on the y-axis (M) was plotted against the mean log2 expression on the x-axis (A). Click here for file

Additional file 8 **PCA plots of B-cell subsets. A-E PCA from the gene expression data set generated from the sorted B-cell subsets.** Each dot represents a sample including PreBI dark green, PreBII salmon pink, immature (I) light green, naive (N) blue, centroblasts (CB) light pink, centrocytes (CC) pink, memory: (M) red; (M_IgM) light red; (M_IgG) red, plasmablasts (PB) and plasma cells (PC) yellow. In PBMNC, cells were either sorted within the same day as purification, depicted with a circle (F), or cryopreserved before sorting, illustrated with a triangle (C). Each of the B cell subpopulations is within two standard deviations from the mean group, illustrated by the ellipses. Click here for file

Additional file 9 **Biological validation in tonsils on the U133 array. Figure S1.** Global GEP data generated from normal tonsil samples on the U133 array. The box plots of eight genes are presented. N: naive B-cells, CB: centroblasts, CC: centrocytes, M: memory B-cells, PB: plasmablasts. *p = 0.002, **p < 0.001. Click here for file

Additional file 10 **Gene specific B-cell atlas in BM. Significantly up-regulated genes in normal B-cell subsets from BM were generated by a 2-way ANOVA model, including donor and B-cell subsets.** Up-regulated genes in each B-cell subset compared to the rest were created (fold change > 2, FDR p-values below 0.05). The top 50 from this list are presented in accordance to p-value. (XLSX 39 kb) Click here for file

Additional file 11 **Gene specific B-cell atlas in Tonsils. Significantly up-regulated genes in normal B-cell subsets from tonsils were generated by a 2-way ANOVA model, including donor and B-cell subsets.** Up-regulated genes in each B-cell subset compared to the rest were created (fold change > 2, FDR p-values below 0.05). The top 50 from this list are presented in accordance to p-value. Click here for file
