# Supplementary material for: Validation and implementation of a method for microarray gene expression profiling of minor B-cell subpopulations in man
Source: BMC Immunol. 2014 Jan 31;15:3. doi: 10.1186/1471-2172-15-3 (PMC3937209; doi:10.1186/1471-2172-15-3)
Supplement: Additional file 1 — Flow chart. Procedure and methods used for establishing GEP from FACS-sorted B-cells on the Exon or U133 array. This included steps for cell sorting, cell lysis/stabilization and storage, RNA isolation, RNA concentration and amplification for microarray analysis. In addition, the CD marker combinations used to distinguish between the B-cell subsets are included. [file 1471-2172-15-3-S1.docx]

**Additional file 1 – Flow chart**

**Isolate and sort cells**

FACSAria cell sorter, BD Biosciences

**mRNA isolation**

µMACS technology, Miltenyi Biotec

**Amplification (input 5µl)**

Ovation pico WTA system, NuGEN

**Concentrate mRNA**

SpeedVac Concentrator, Eppendorf

**Purify amplified anti-sence cDNA**

QIAquick PCR purification, Qiagen

**Exon array only: Convert to**

**sence cDNA (input 3µg)**

WT-Ovation exon, NuGEN

(WT-Ovation Exon module)

**Fragmentation/labelling (input 5 µg)**

Encore biotin, NuGEN

**Hybridization to gene chip array**

Human Exon 1.0 ST or HG U133 Plus 2.0

Affymetrix

Selection of storage buffer for sorted cells

Gene specific amplification determined by qPCR

Comparing NuGEN protocol to standard protocol

Concordance between the pre-defined CD marker and array-based transcript expression

Biological validation

Amplified yield from FACS tonsil B-cell subsets

Tissue-independent yield and QC on microarray data

**Experiments conducted**


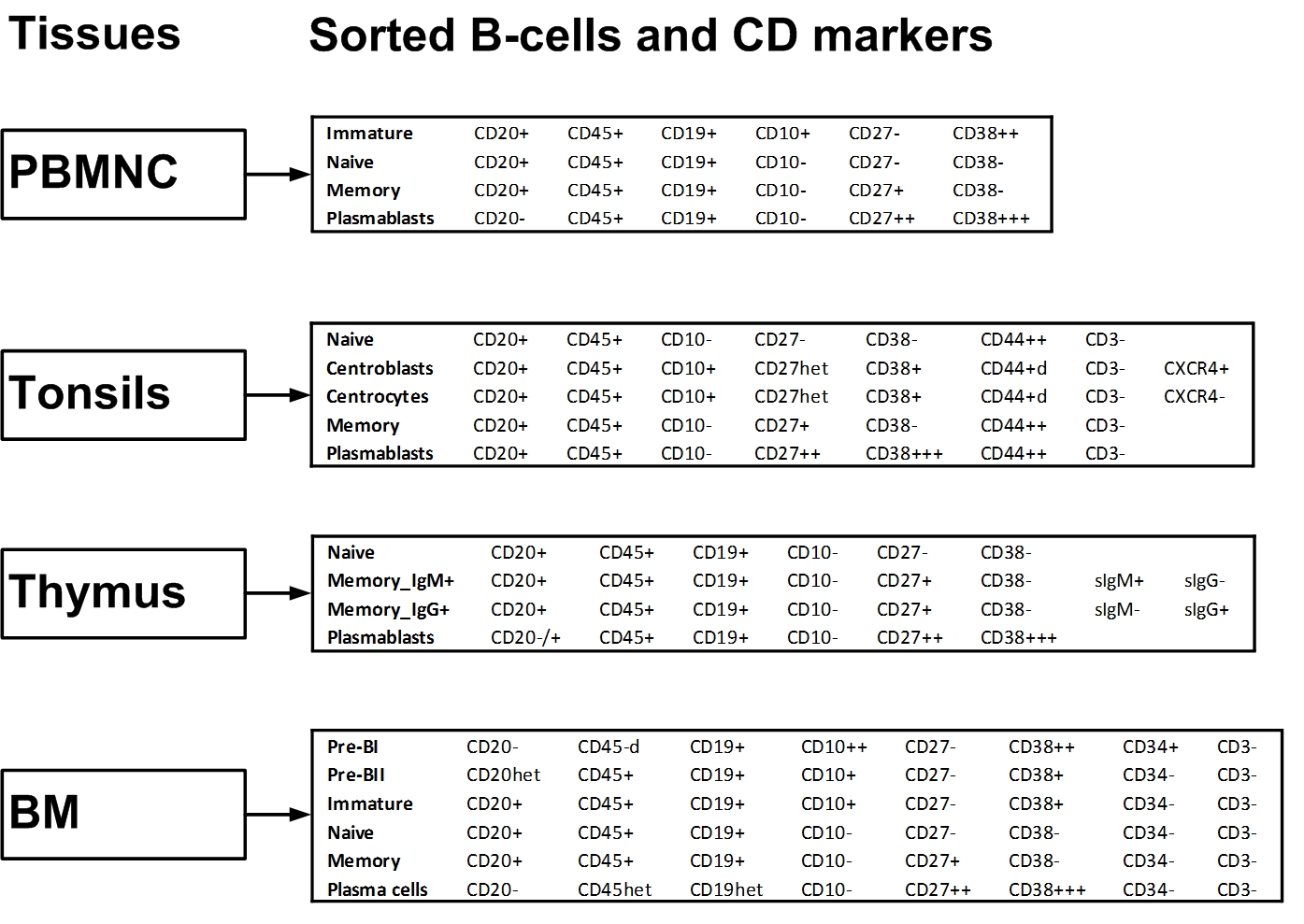


B-cell subsets in PBMNC

| • Immature | CD20+ | CD45+ | CD19+ | CD10+ | CD27- | CD38++ |
| --- | --- | --- | --- | --- | --- | --- |
| • Naive | CD20+ | CD45+ | CD19+ | CD10- | CD27- | CD38- |
| • Memory | CD20+ | CD45+ | CD19+ | CD10- | CD27+ | CD38- |
| • Plasmablasts | CD20- | CD45+ | CD19+ | CD10- | CD27++ | CD38+++ |

B-cell subsets in tonsil

| • Naive | CD20+ | CD45+ | CD10- | CD27- | CD38- | CD44++ | CD3- |  |
| --- | --- | --- | --- | --- | --- | --- | --- | --- |
| • Centroblasts | CD20+ | CD45+ | CD10+ | CD27het | CD38+ | CD44 + d | CD3- | CXCR4+ |
| • Ccentrocytes | CD20+ | CD45+ | CD10+ | CD27het | CD38+ | CD44 + d | CD3- | CXCR4- |
| • Memory | CD20+ | CD45+ | CD10- | CD27+ | CD38- | CD44++ | CD3- |  |
| • Plasmablasts | CD20+ | CD45+ | CD10- | CD27++ | CD38+++ | CD44++ | CD3- |  |

B-cell subsets in thymus

| • Naive | CD20+ | CD45+ | CD19+ | CD10- | CD27- | CD38- |  |  |
| --- | --- | --- | --- | --- | --- | --- | --- | --- |
| • Memory_IgM+ | CD20+ | CD45+ | CD19+ | CD10- | CD27+ | CD38- | sIgM+ | sIgG- |
| • Memory_IgG+ | CD20+ | CD45+ | CD19+ | CD10- | CD27+ | CD38- | sIgM- | sIgG+ |
| • Plasmablasts | CD20−/+ | CD45+ | CD19+ | CD10- | CD27++ | CD38+++ |  |  |

B-cell subsets in BM

| • Pre-BI | CD20- | CD45-d | CD19+ | CD10++ | CD27- | CD38++ | CD34+ | CD3- |
| --- | --- | --- | --- | --- | --- | --- | --- | --- |
| • Pre-BII | CD20het | CD45+ | CD19+ | CD10+ | CD27- | CD38+ | CD34- | CD3- |
| • Immature | CD20+ | CD45+ | CD19+ | CD10+ | CD27- | CD38+ | CD34- | CD3- |
| • Naive | CD20+ | CD45+ | CD19+ | CD10- | CD27- | CD38- | CD34- | CD3- |
| • Memory | CD20+ | CD45+ | CD19+ | CD10- | CD27+ | CD38- | CD34- | CD3- |
| • Plasma cells | CD20- | CD45het | CD19het | CD10- | CD27++ | CD38+++ | CD34- | CD3- |
