# Supplementary material for: Validation and implementation of a method for microarray gene expression profiling of minor B-cell subpopulations in man
Source: BMC Immunol. 2014 Jan 31;15:3. doi: 10.1186/1471-2172-15-3 (PMC3937209; doi:10.1186/1471-2172-15-3)
Supplement: Additional file 2 — Initial data analysis of quality. Table S1. Initial data analysis of the quality and technical performance of the microarrays was performed using the AGCC. For the tonsils on the U133 arrays, .CEL-files were normalized with MAS5.0. Scaling factor (SF), percentage of present calls (%P), and signal ratios of probe sets interrogating different segments (3′/5′) were comparable across samples, with similar variation among samples of the various B-cell subsets. For the Exon arrays, .CEL-files were normalized with PLIER and %P was evaluated for each tissue ( PBMNC (fresh) mean 53 ± 7; %P thymus mean 51 ± 5; %P BM mean 50 ± 6). Further analysis was performed by evaluating the histogram and signal box plots of the signals. [file 1471-2172-15-3-S2.docx]

**Additional file 2 – initial data analysis of quality**

Tabel 1

| **Subset** | **SF** | **%P** | **β-Actin 3/5** | **GAPDH 3/5** |
| --- | --- | --- | --- | --- |
| Naïve | 3.0 | 50 | 1.8 | 1.1 |
| Naïve | 4.5 | 41 | 1.6 | 1.0 |
| Naïve | 4.7 | 44 | 2.1 | 1.0 |
| Naïve | 5.2 | 42 | 1.7 | 2.0 |
| Naïve | 4.0 | 46 | 1.6 | 1.2 |
| Naïve | 3.7 | 49 | 1.8 | 1.1 |
| Centrocytes | 2.6 | 53 | 1.6 | 0.9 |
| Centrocytes | 3.1 | 51 | 1.7 | 1.0 |
| Centrocytes | 3.8 | 51 | 2.1 | 1.1 |
| Centrocytes | 4.0 | 47 | 1.8 | 1.0 |
| Centrocytes | 4.2 | 50 | 2.0 | 1.0 |
| Centrocytes | 4.1 | 50 | 1.6 | 1.0 |
| Centrocytes | 3.8 | 49 | 1.5 | 1.1 |
| Centroblasts | 2.8 | 52 | 1.6 | 0.9 |
| Centroblasts | 3.3 | 52 | 1.8 | 0.9 |
| Centroblasts | 3.9 | 49 | 1.7 | 1.0 |
| Centroblasts | 3.6 | 49 | 1.7 | 1.0 |
| Centroblasts | 3.6 | 52 | 2.0 | 1.0 |
| Centroblasts | 3.7 | 52 | 2.4 | 1.7 |
| Centroblasts | 3.7 | 47 | 1.7 | 1.3 |
| Memory | 3 | 50 | 1.7 | 0.9 |
| Memory | 7.9 | 41 | 2.0 | 1.0 |
| Memory | 5.2 | 41 | 2.0 | 1.0 |
| Memory | 5.8 | 45 | 1.7 | 1.0 |
| Memory | 4.0 | 48 | 1.7 | 0.8 |
| Memory | 4.3 | 47 | 1.7 | 0.8 |
| Plasmablasts | 2.9 | 54 | 3.0 | 0.8 |
| Plasmablasts | 3.6 | 52 | 4.5 | 0.9 |
| Plasmablasts | 4.5 | 51 | 6.5 | 0.9 |
| Plasmablasts | 4.9 | 50 | 5.8 | 0.9 |
| Plasmablasts | 4.3 | 49 | 4.6 | 1.0 |
| Plasmablasts | 4.4 | 51 | 4.7 | 1.0 |
| Plasmablasts | 4.1 | 51 | 6.0 | 1.0 |
| Average | 4.1 | 49 | 2.5 | 1.0 |
| SD | 1.0 | 4 | 1.4 | 0.2 |
