# Supplementary material for: Validation and implementation of a method for microarray gene expression profiling of minor B-cell subpopulations in man
Source: BMC Immunol. 2014 Jan 31;15:3. doi: 10.1186/1471-2172-15-3 (PMC3937209; doi:10.1186/1471-2172-15-3)
Supplement: Additional file 3 — Concordance between the pre-defined CD marker and array based transcript expressions. The B-cell subsets were divided into two groups based on the pre-defined positive or negative CD marker. The mean and standard deviations were calculated for the positive and negative groups based on the gene expression value for the CD marker. Discordance was noticed if the gene expression values for a B-cell subset in the opposite group as a pre-defined CD marker. Fisher’s exact test for 2x2 tables was used to test for independence between the groupings based on CD marker and GEP. Table S1. Concordance between the pre-defined CD markers and transcript expression on array in BM. Table S2. Concordance between the pre-defined CD markers and transcript expression on array in PBMNC. Table S3. Concordance between the pre-defined CD markers and transcript expression on array in thymus. [file 1471-2172-15-3-S3.docx]

**Additional file 3 - Concordance between the pre-defined CD marker and array based transcript expressions**

Table 1

| **CD38** | | **GEP** | |  |
| --- | --- | --- | --- | --- |
|  |  | **+** | **-** | **Total** |
| **CD** | **+** | 23 | 0 | 23 |
|  | **-** | 0 | 13 | 13 |
|  | **Total** | 23 | 13 | 36 |

p << 0.001

| **CD34** | | **GEP** | |  |
| --- | --- | --- | --- | --- |
|  |  | **+** | **-** | **Total** |
| **CD** | **+** | 6 | 0 | 6 |
|  | **-** | 0 | 30 | 30 |
|  | **Total** | 6 | 30 | 36 |

p << 0.001

| **CD10** | | **GEP** | |  |
| --- | --- | --- | --- | --- |
|  |  | **+** | **-** | **Total** |
| **CD** | **+** | 17 | 0 | 17 |
|  | **-** | 2 | 17 | 19 |
|  | **Total** | 19 | 17 | 36 |

p << 0.001

| **CD27** | | **GEP** | |  |
| --- | --- | --- | --- | --- |
|  |  | **+** | **-** | **Total** |
| **CD** | **+** | 12 | 0 | 12 |
|  | **-** | 0 | 24 | 24 |
|  | **Total** | 12 | 24 | 36 |

p << 0.001

| **CD20** | | **GEP** | |  |
| --- | --- | --- | --- | --- |
|  |  | **+** | **-** | **Total** |
| **CD** | **+** | 17 | 1 | 18 |
|  | **-** | 0 | 18 | 18 |
|  | **Total** | 17 | 19 | 36 |

p << 0.001

Table 2

| **CD38** | | **GEP** | |  |
| --- | --- | --- | --- | --- |
|  |  | **+** | **-** | **Total** |
| **CD** | **+** | 22 | 0 | 22 |
|  | **-** | 3 | 19 | 22 |
|  | **Total** | 25 | 19 | 44 |

p << 0.001

| **CD10** | | **GEP** | |  |
| --- | --- | --- | --- | --- |
|  |  | **+** | **-** | **Total** |
| **CD** | **+** | 12 | 0 | 12 |
|  | **-** | 1 | 31 | 32 |
|  | **Total** | 13 | 31 | 44 |

p << 0.001

| **CD27** | | **GEP** | |  |
| --- | --- | --- | --- | --- |
|  |  | **+** | **-** | **Total** |
| **CD** | **+** | 22 | 0 | 22 |
|  | **-** | 2 | 20 | 22 |
|  | **Total** | 24 | 20 | 44 |

p << 0.001

| **CD20** | | **GEP** | |  |
| --- | --- | --- | --- | --- |
|  |  | **+** | **-** | **Total** |
| **CD** | **+** | 34 | 0 | 34 |
|  | **-** | 1 | 9 | 10 |
|  | **Total** | 35 | 9 | 44 |

p << 0.001

Table 3

| **CD27** | | **GEP** | |  |
| --- | --- | --- | --- | --- |
|  |  | **+** | **-** | **Total** |
| **CD** | **+** | 14 | 0 | 14 |
|  | **-** | 0 | 4 | 4 |
|  | **Total** | 14 | 4 | 18 |

p << 0.001

| **CD38** | | **GEP** | |  |
| --- | --- | --- | --- | --- |
|  |  | **+** | **-** | **Total** |
| **CD** | **+** | 5 | 0 | 5 |
|  | **-** | 1 | 12 | 13 |
|  | **Total** | 6 | 12 | 18 |

p << 0.001

| **CD20** | | **GEP** | |  |
| --- | --- | --- | --- | --- |
|  |  | **+** | **-** | **Total** |
| **CD** | **+** | 15 | 0 | 15 |
|  | **-** | 0 | 3 | 3 |
|  | **Total** | 15 | 3 | 18 |

p << 0.001
