# Supplementary material for: Validation and implementation of a method for microarray gene expression profiling of minor B-cell subpopulations in man
Source: BMC Immunol. 2014 Jan 31;15:3. doi: 10.1186/1471-2172-15-3 (PMC3937209; doi:10.1186/1471-2172-15-3)
Supplement: Additional file 4 — Fidelity of amplification. Table S1. Six CCLs were ranked from high to low expression and normalised to GAPDH. This ranking was performed for both the non-amplified and the amplified CCLs, and compared using Spearman’s rank correlation. A test for inconsistent ranking was carried out by an exact permutation test. [file 1471-2172-15-3-S4.docx]

**Additional file 4 - Fidelity of amplification**

**Table 1**

| **KMM-1** ( ΔCq = Cq_gene_ - Cq_GAPDH_)  p = 0.02 | | |
| --- | --- | --- |
| **Rank** | **Non-amplified** | **Amplified** |
| 1-1 | PPIA (-0.30) | PPIA (-5.74) |
| 2-3 | XBP1 (2.32) | PRDM1 (-0.45) |
| 3-2 | PRDM1 (4.78) | XBP1 (-0.22) |
| 4-5 | MGST1 (5.30) | IRF4 (1.31) |
| 5-7 | WHSC1 (5.65) | MGST1 (2.89) |
| 6-4 | IRF4 (6.56) | TBP (3.99) |
| 7-6 | TBP (7.16) | WHSC1 (5.79) |

| **KMS-12-BM** (ΔCq = Cq_gene_ - Cq_GAPDH_)  p = 0.10 | | |
| --- | --- | --- |
| **Rank** | **Non-amplified** | **Amplified** |
| 1-1 | PPIA (-1.78) | PPIA (-6.62) |
| 2-4 | XBP1 (4.34) | IRF4 (1.40) |
| 3-6 | WHSC1 (5.33) | PRDM1 (1.73) |
| 4-3 | PRDM1 (5.74) | XBP1 (2.14) |
| 5-2 | IRF4 (6.20) | TBP (2.86) |
| 6-5 | TBP (6.31) | WHSC1 (5.39) |
| 7-7 | MGST1 (7.21) | MGST1 (5.50) |

| **LP-1** (ΔCq = Cq_gene_ - Cq_GAPDH_)  p = 0.05 | | |
| --- | --- | --- |
| **Rank** | **Non-amplified** | **Amplified** |
| 1-1 | PPIA (-0.12) | PPIA (-7.42) |
| 2-3 | XBP1 (2.26) | PRDM1 (-3.16) |
| 3-2 | PRDM1 (3.99) | XBP1 (-2.31) |
| 4-6 | WHSC1 (4.61) | IRF4 (-0.99) |
| 5-4 | IRF4 (5.27) | TBP (2.97) |
| 6-5 | TBP (7.51) | WHSC1 (3.32) |
|  | MGST1 (absent) | MGST1 (absent) |

| **MOLP-8 (**ΔCq = Cq_gene_ - Cq_GAPDH_)  p = 0.01 | | |
| --- | --- | --- |
| **Rank** | **Non-amplified** | **Amplified** |
| 1-1 | PPIA (-0.12) | PPIA (-6.60) |
| 2-2 | XBP1 (1.79) | XBP1 (-2.10) |
| 3-4 | MGST1 (3.97) | PRDM1 (-1.80) |
| 4-3 | PRDM1 (4.50) | MGST1 (-0.13) |
| 5-7 | WHSC1 (4.96) | IRF4 (-0.04) |
| 6-5 | IRF4 (6.50) | TBP (2.93) |
| 7-6 | TBP (7.69) | WHSC1 (4.60) |

| **OPM-2** (ΔCq = Cq_gene_ - Cq_GAPDH_)  p = 0.07 | | |
| --- | --- | --- |
| **Rank** | **Non-amplified** | **Amplified** |
| 1-1 | PPIA (-0.24) | PPIA (-5.92) |
| 2-3 | XBP1 (2.76) | PRDM1 (-1.79) |
| 3-5 | WHSC1 (4.10) | XBP1 (0.02) |
| 4-2 | PRDM1 (4.45) | IRF4 (1.23) |
| 5-4 | IRF4 (6.91) | WHSC1 (2.98) |
| 6-6 | TBP (8.14) | TBP (3.69) |
|  | MGST1 (absent) | MGST1 (absent) |

| **RPMI-8226** (ΔCq = Cq_gene_ - Cq_GAPDH_)  p = 0.03 | | |
| --- | --- | --- |
| **Rank** | **Non-amplified** | **Amplified** |
| 1-1 | PPIA (-0.83) | PPIA (-6.57) |
| 2-3 | XBP1 (4.06) | PRDM1 (0.05) |
| 3-4 | MGST1 (4.47) | XBP1 (1.19) |
| 4-2 | PRDM1 (5.72) | MGST1 (1.48) |
| 5-7 | WHSC1 (6.02) | IRF4 (2.14) |
| 6-6 | TBP (7.69) | TBP (4.27) |
| 7-5 | IRF4 (7.83) | WHSC1 (6.67) |
