# Supplementary figures and images for: Validation and implementation of a method for microarray gene expression profiling of minor B-cell subpopulations in man
Source: BMC Immunol. 2014 Jan 31;15:3. doi: 10.1186/1471-2172-15-3 (PMC3937209; doi:10.1186/1471-2172-15-3)

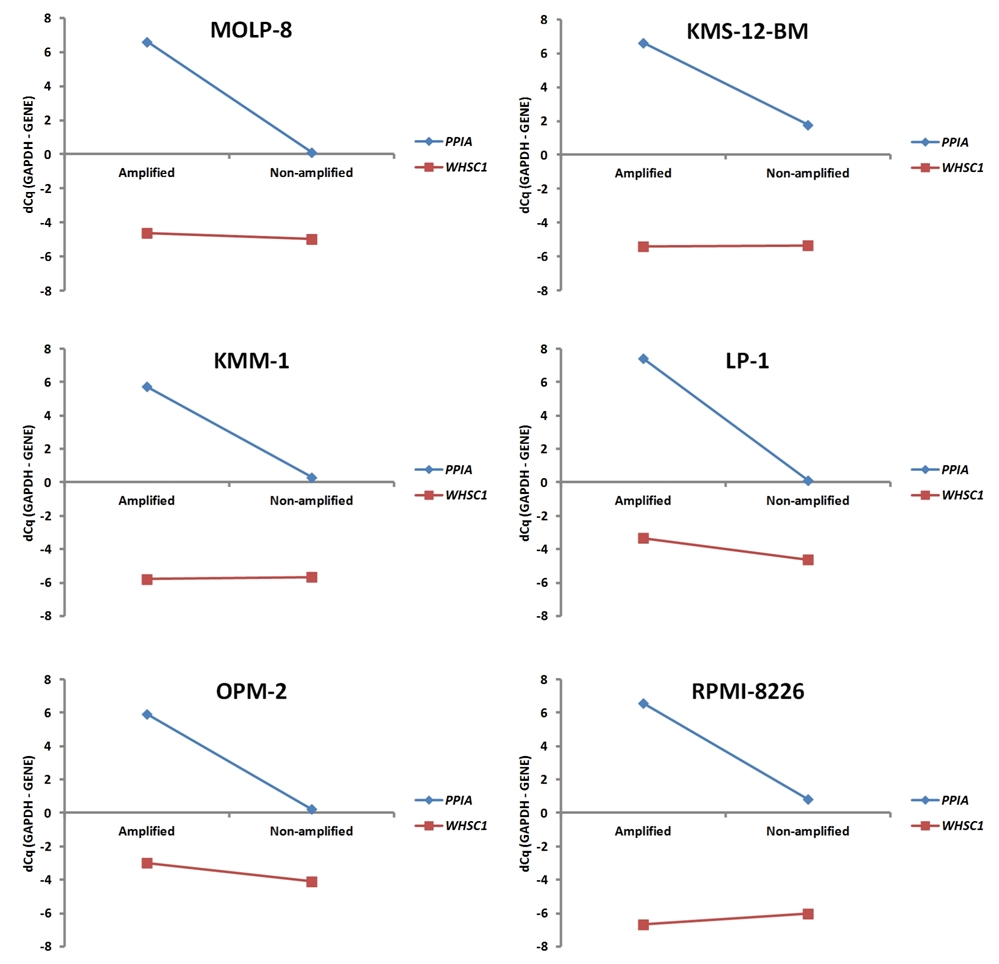

Supplement: Additional file 5 — PPIA and WHSC1 were normalised to GAPDH and the gene expression is shown in six non-amplified and amplified CCLs. The amplification method induces a sequence specific bias, resulting in some sequences or parts of transcripts amplify better than others like PPIA compared to WHSC1. However, this amplification bias for a gene is preserved across the CCLs. [file 1471-2172-15-3-S5.jpeg]

**Additional file 7 - Reproducibility between NuGEN and Ambion protocol**

**Figure 1**

**
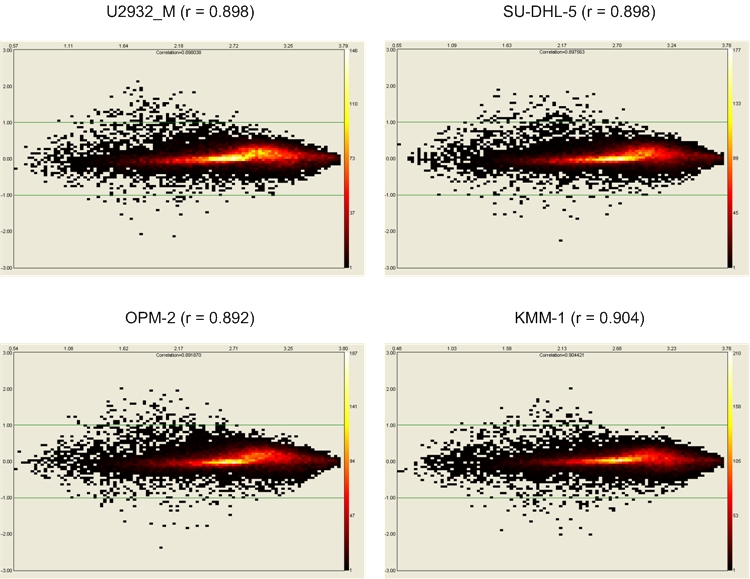
**

Supplement: Additional file 7 — Reproducibility between NuGEN and Ambion protocol. Figure S1. MA plots were generated for the pair-wise comparisons between CCLs subjected to the NuGEN and Ambion protocol. Exon array signal values were normalized using RMA and Pearson’s correlation coefficient was calculated using the Affymetrix Expression Console analysis package. The log2 fold change on the y-axis (M) was plotted against the mean log2 expression on the x-axis (A). [file 1471-2172-15-3-S7.docx]
