# Supplementary material for: Validation and implementation of a method for microarray gene expression profiling of minor B-cell subpopulations in man
Source: BMC Immunol. 2014 Jan 31;15:3. doi: 10.1186/1471-2172-15-3 (PMC3937209; doi:10.1186/1471-2172-15-3)
Supplement: Additional file 6 — Expression of selected genes between two protocols on the Exon array. Table S1. Expression value of seven selected genes between NuGen and the Ambion protocol on the Exon array. RNA was extracted from the same four CCL (KMM-1, OPM-2, SU-DHL-5 and U2932) and a 100 times less RNA was used as input in the NuGEN protocol compared to Ambion protocol. [file 1471-2172-15-3-S6.docx]

**Additional file 6 - Expression of selected genes between two protocols on the Exon array**

**Table 1**

| **IRF4** | **NuGEN** | **Ambion** |
| --- | --- | --- |
| KMM-1 | 11.5 | 11.4 |
| OPM-2 | 11.4 | 11.7 |
| SU-DHL-5 | 6.8 | 5.8 |
| U2932_M | 10.6 | 10.9 |
|  |  |  |
| **PRDM1** | **NuGEN** | **Ambion** |
| KMM-1 | 10.8 | 10.9 |
| OPM-2 | 11.0 | 11.5 |
| SU-DHL-5 | 6.7 | 5.9 |
| U2932_M | 8.6 | 9.1 |
|  |  |  |
| **XBP1** | **NuGEN** | **Ambion** |
| KMM-1 | 11.0 | 12.2 |
| OPM-2 | 10.7 | 11.8 |
| SU-DHL-5 | 7.2 | 8.8 |
| U2932_M | 9.6 | 11.0 |
|  |  |  |
| **TBP** | **NuGEN** | **Ambion** |
| KMM-1 | 8.9 | 9.5 |
| OPM-2 | 8.5 | 9.4 |
| SU-DHL-5 | 8.1 | 9.3 |
| U2932_M | 8.2 | 8.5 |
|  |  |  |
| **PPIA** | **NuGEN** | **Ambion** |
| KMM-1 | 7.5 | 7.7 |
| OPM-2 | 7.2 | 7.4 |
| SU-DHL-5 | 7.7 | 7.7 |
| U2932_M | 7.5 | 7.9 |
|  |  |  |
| **WHSC1** | **NuGEN** | **Ambion** |
| KMM-1 | 8.4 | 8.0 |
| OPM-2 | 9.9 | 9.8 |
| SU-DHL-5 | 8.7 | 8.4 |
| U2932_M | 8.5 | 8.0 |
|  |  |  |
| **MGST1** | **NuGEN** | **Ambion** |
| KMM-1 | 7.0 | 8.2 |
| OPM-2 | 3.7 | 3.2 |
| SU-DHL-5 | 3.6 | 3.3 |
| U2932_M | 7.5 | 9.5 |
