# Supplementary material for: Validation and implementation of a method for microarray gene expression profiling of minor B-cell subpopulations in man
Source: BMC Immunol. 2014 Jan 31;15:3. doi: 10.1186/1471-2172-15-3 (PMC3937209; doi:10.1186/1471-2172-15-3)
Supplement: Additional file 8 — PCA plots of B-cell subsets. A-E PCA from the gene expression data set generated from the sorted B-cell subsets. Each dot represents a sample including PreBI dark green, PreBII salmon pink, immature (I) light green, naive (N) blue, centroblasts (CB) light pink, centrocytes (CC) pink, memory: (M) red; (M_IgM) light red; (M_IgG) red, plasmablasts (PB) and plasma cells (PC) yellow. In PBMNC, cells were either sorted within the same day as purification, depicted with a circle (F), or cryopreserved before sorting, illustrated with a triangle (C). Each of the B cell subpopulations is within two standard deviations from the mean group, illustrated by the ellipses. [file 1471-2172-15-3-S8.docx]

**Additional file 8 – PCA plots of B-cell subsets**

**A: BM**


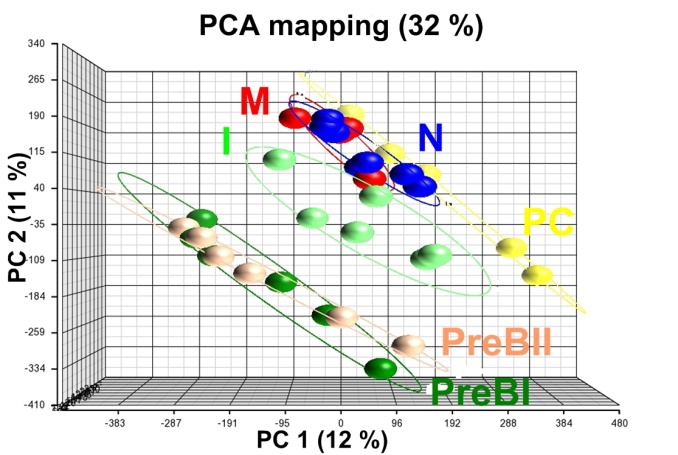

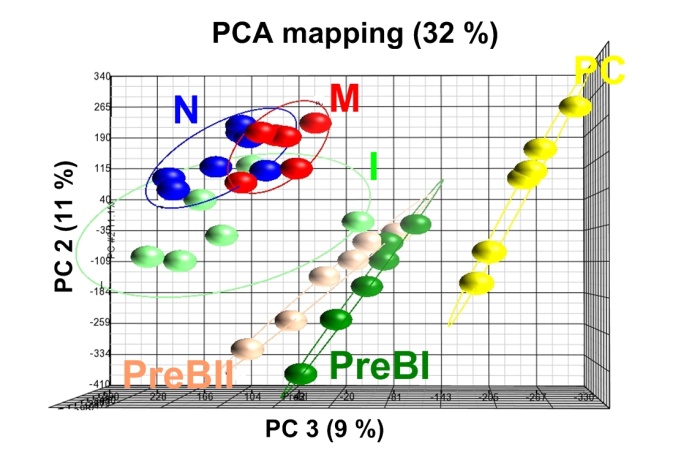


**B: PBMNC (fresh and cryopreserved)**

**
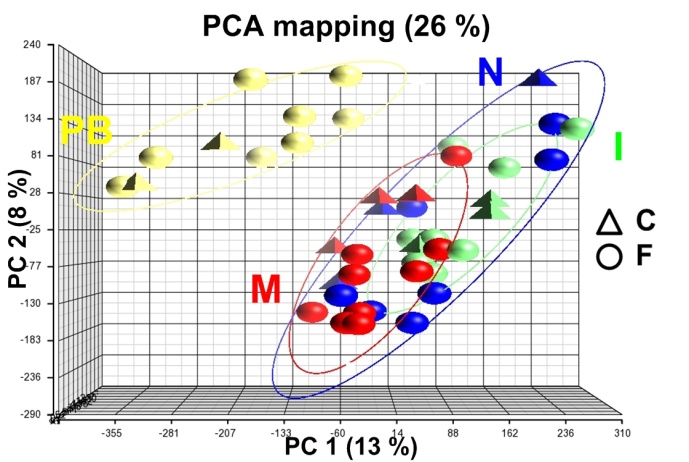

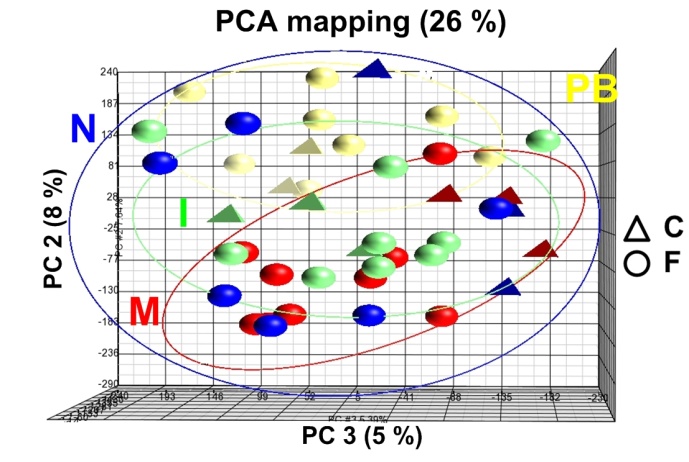
**

**C: Tonsils (Exon array)**

**
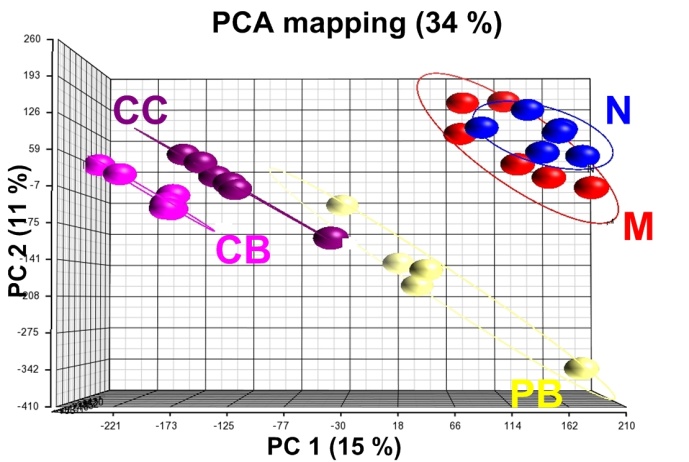

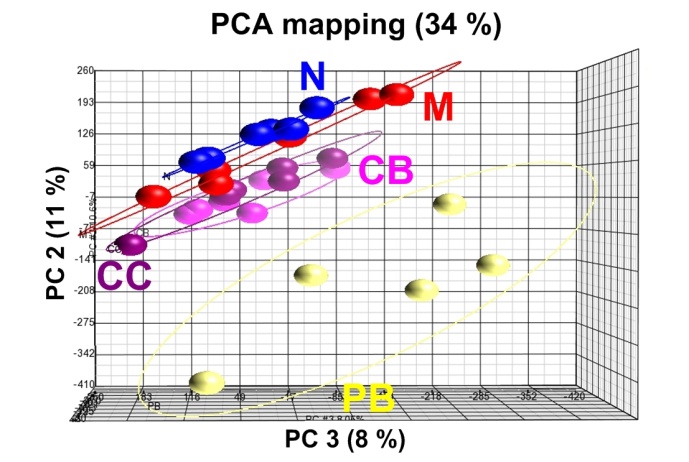
**

**D: Tonsils (U133 array)**

**
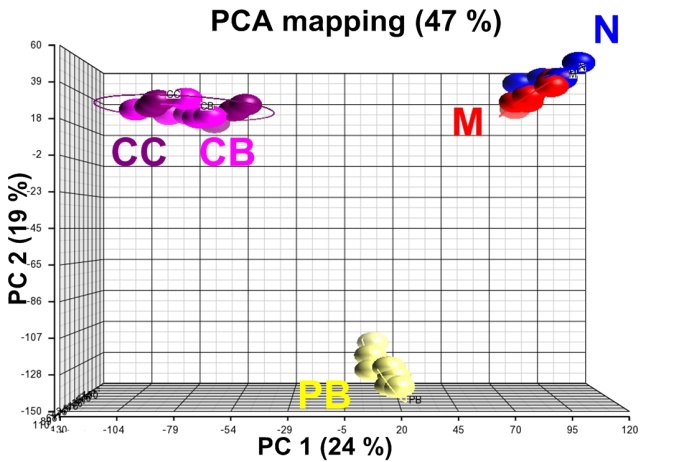

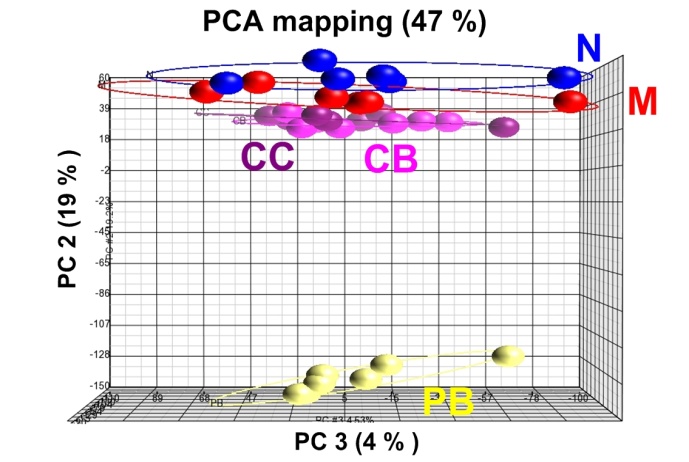
**

**E: Thymus**

**
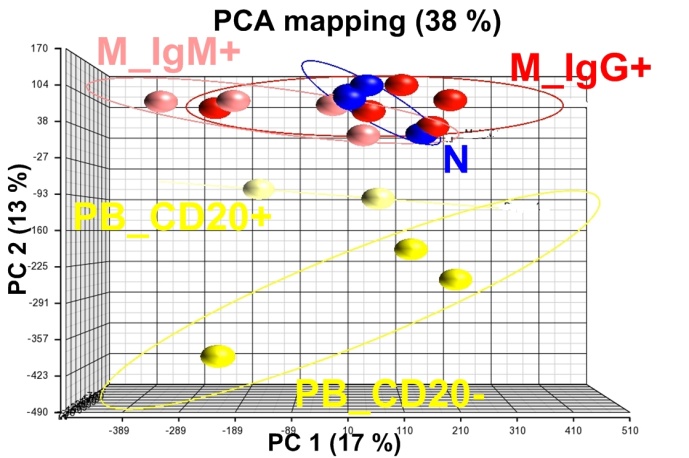
 *
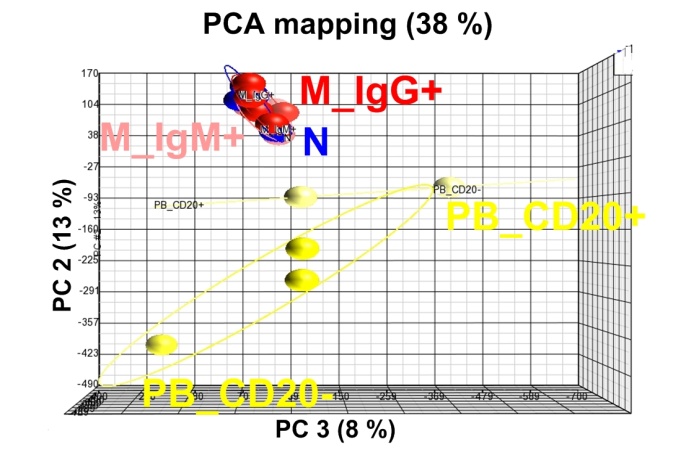
***
