# Supplementary material for: Validation and implementation of a method for microarray gene expression profiling of minor B-cell subpopulations in man
Source: BMC Immunol. 2014 Jan 31;15:3. doi: 10.1186/1471-2172-15-3 (PMC3937209; doi:10.1186/1471-2172-15-3)
Supplement: Additional file 9 — Biological validation in tonsils on the U133 array. Figure S1. Global GEP data generated from normal tonsil samples on the U133 array. The box plots of eight genes are presented. N: naive B-cells, CB: centroblasts, CC: centrocytes, M: memory B-cells, PB: plasmablasts. *p = 0.002, **p < 0.001. [file 1471-2172-15-3-S9.docx]

**Additional file 9 - Biological validation in tonsils on the U133 array**

**Figure 1**

**
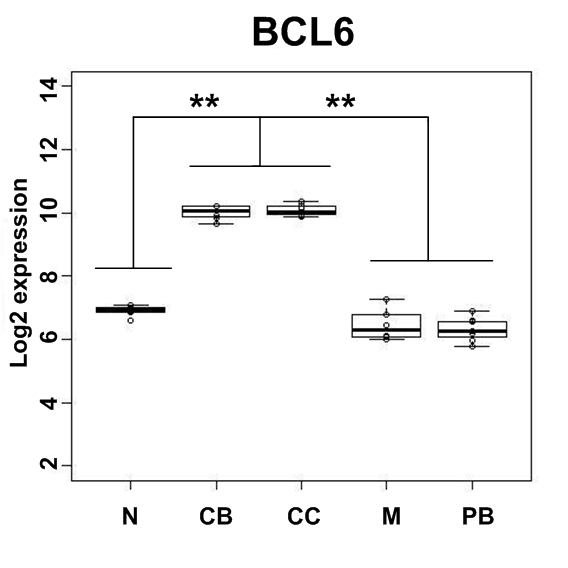

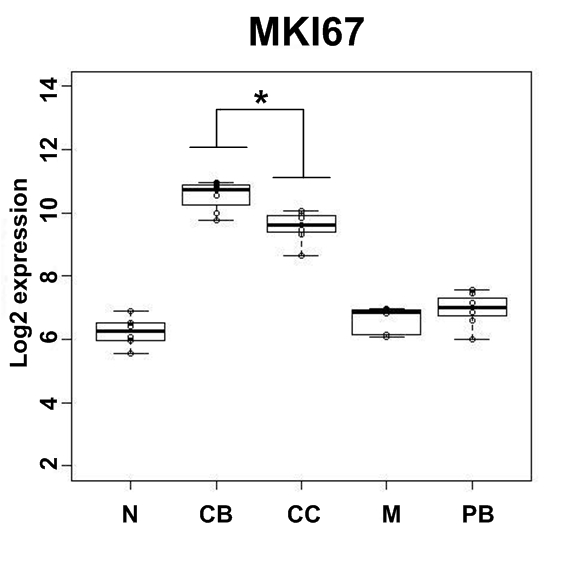
**

**
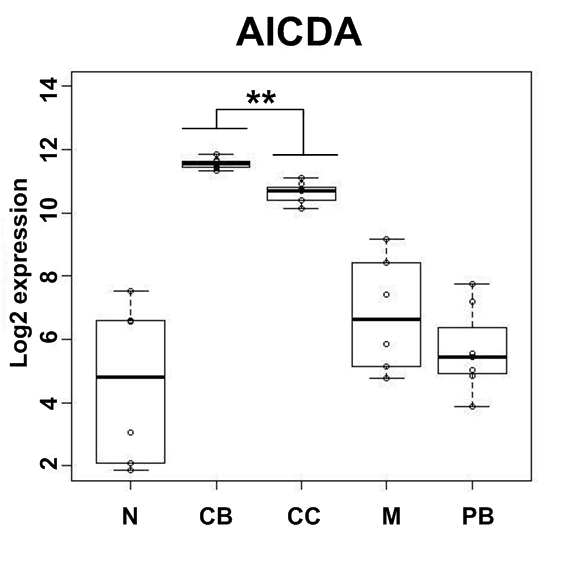

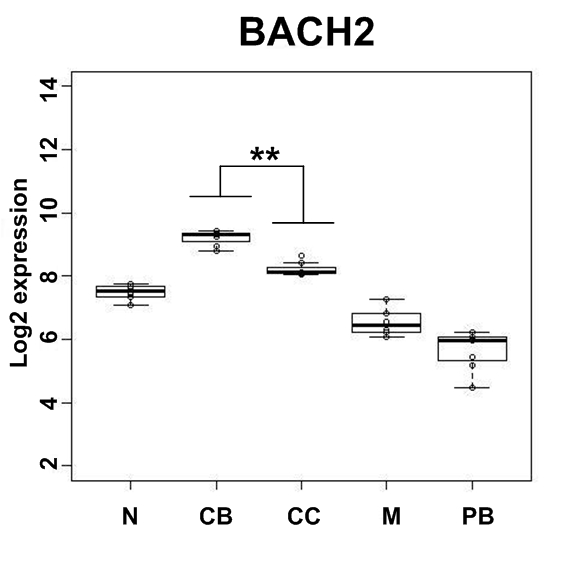
**

**
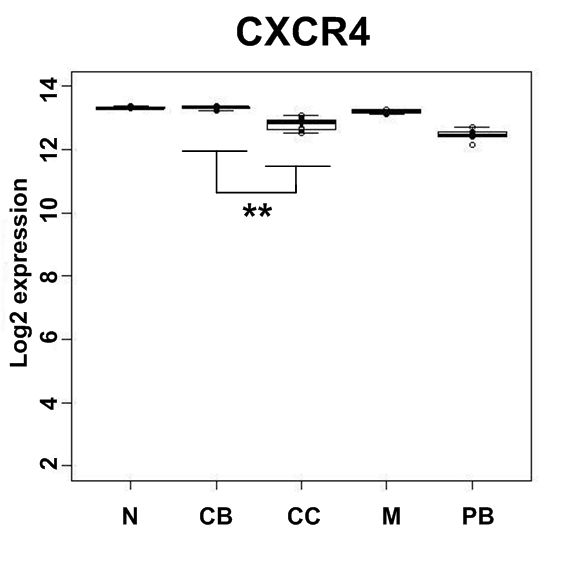

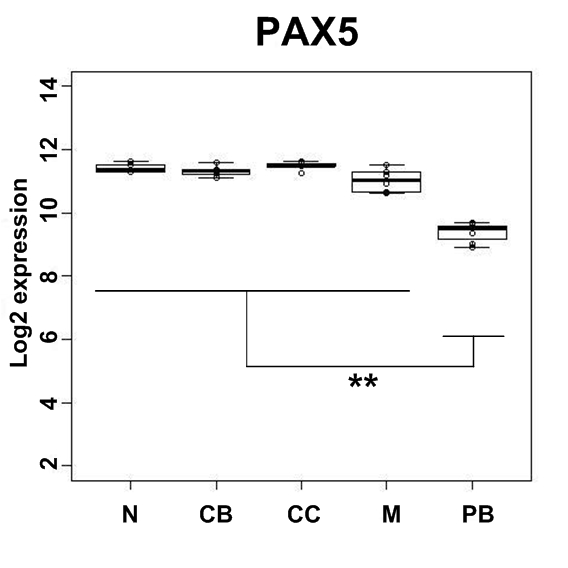
**

**
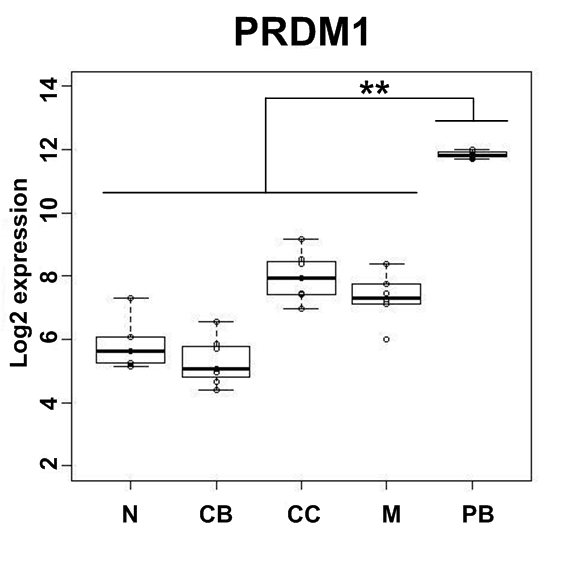

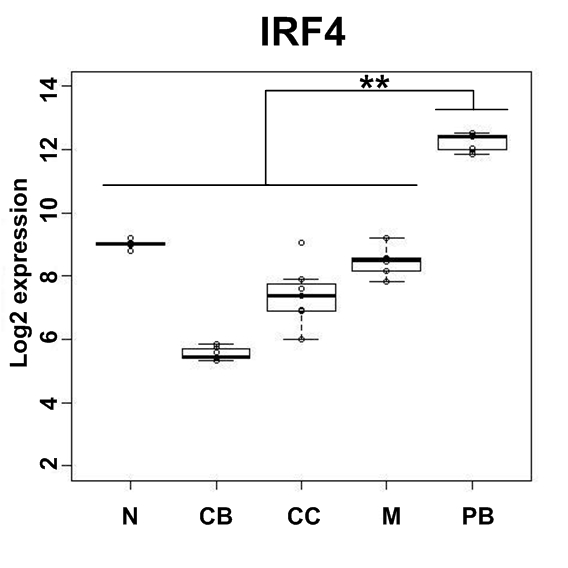
**

BCL6 is the master regulator of the GC reaction [1] and were significantly up-regulated in CB and CC compared to the other B-cell subsets.

MKI67 is a proliferation marker and were significantly up-regulated in CB compared to CC as expected [2,3].

AICDA involved in somatic hypermutation (SHM) and isotype class switch recombination (CSR) in GC B-cells [4] were significantly up-regulated in CB and CC compared to the other B-cell subsets. In addition, AICDA were significantly higher expressed in CB compared to CC.

BACH2 required for SHM and CSR [1] were significantly up-regulated in CB and CC compared to the other B-cell subsets. In addition, BACH2 were significantly higher expressed in CB compared to CC.

CXCR4 used to discriminate between CB and CC in the FACS purification step was significantly higher expressed in CB.

PAX5, which is essential for early commitment to the B-cell lineage and maintained in all B-cell subsets, except in PB/PC [5], was significantly up-regulated in the four B-cell subsets compared to the PB.

The TFs PRDM1 and XBP1, which promote and facilitate PC differentiation [6], were significantly up-regulated in PC.

**Reference List**

**1. Schmidlin H, Diehl SA, Blom B: New insights into the regulation of human B-cell differentiation. *Trends Immunol* 2009, 30: 277-285.**

**2. Caron G, Le GS, Lamy T, Tarte K, Fest T: CXCR4 expression functionally discriminates centroblasts versus centrocytes within human germinal center B cells. *J Immunol* 2009, 182: 7595-7602.**

**3. Victora GD, Schwickert TA, Fooksman DR, Kamphorst AO, Meyer-Hermann M, Dustin ML *et al*.: Germinal center dynamics revealed by multiphoton microscopy with a photoactivatable fluorescent reporter. *Cell* 2010, 143: 592-605.**

**4. Gazumyan A, Bothmer A, Klein IA, Nussenzweig MC, McBride KM: Activation-induced cytidine deaminase in antibody diversification and chromosome translocation. *Adv Cancer Res* 2012, 113: 167-190.**

**5. Cattoretti G, Shaknovich R, Smith PM, Jack HM, Murty VV, Alobeid B: Stages of germinal center transit are defined by B cell transcription factor coexpression and relative abundance. *J Immunol* 2006, 177: 6930-6939.**

**6. Matthias P, Rolink AG: Transcriptional networks in developing and mature B cells. *Nat Rev Immunol* 2005, 5: 497-508.**
